# MHD mixed convective stagnation point flow of nanofluid past a permeable stretching sheet with nanoparticles aggregation and thermal stratification

**DOI:** 10.1038/s41598-022-20074-1

**Published:** 2022-09-26

**Authors:** Zafar Mahmood, Sharifah E. Alhazmi, Awatif Alhowaity, Riadh Marzouki, Nadir Al-Ansari, Umar Khan

**Affiliations:** 1 Department of Mathematics and Statistics, Hazara University, Mansehra, Pakistan; 2 Mathematics Department, Al-Qunfudah University College, Umm Al-Qura University, Mecca, Kingdom of Saudi Arabia; 3 Department of Mathematics, College of Science and Arts at Alkamil, University of Jeddah, Jeddah, Saudi Arabia; 4 Chemistry Department, College of Science, King Khalid University, Abha, 61413 Saudi Arabia; 5 Chemistry Department, Faculty of Sciences of Sfax, University of Sfax, 3038 Sfax, Tunisia; 6 Department of Civil, Environmental and Natural Resources Engineering, Lulea University of Technology, 97187 Lulea, Sweden

**Keywords:** Energy science and technology, Materials science, Mathematics and computing, Nanoscience and technology

## Abstract

Using a thermally stratified water-based nanofluid and a permeable stretching sheet as a simulation environment, this research examines the impact of nanoparticle aggregation on MHD mixed convective stagnation point flow. Nanoparticle aggregation is studied using two modified models: the Krieger–Dougherty and the Maxwell–Bruggeman. The present problem's governing equations were transformed into a solvable mathematical model utilizing legitimate similarity transformations, and numerical solutions were then achieved using shooting with Runge–Kutta Fehlberg (RKF) technique in Mathematica. Equilibrium point flow toward permeable stretching surface is important for the extrusion process because it produces required heat and mass transfer patterns and identifies and clarifies fragmented flow phenomena using diagrams. Nanoparticle volume fraction was shown to have an impact on the solutions' existence range, as well. Alumina and copper nanofluids have better heat transfer properties than regular fluids. The skin friction coefficients and Nusselt number, velocity, temperature profiles for many values of the different parameters were obtained. In addition, the solutions were shown in graphs and tables, and they were explained in detail. A comparison of the current study's results with previous results for a specific instance is undertaken to verify the findings, and excellent agreement between them is observed.

## Introduction

In most cases, a flow that encounters a solid surface and splits into two distinct zones is described as experiencing a "stagnation point." In the industrial and technical sectors, this kind of flow has been widely used because it has the best heat transmission, fluid pressure, and mass deposition rate in the stagnation point zone. References^1,2^ were the first examples of stagnation point flow in ancient literature. Chiam^3^ was the first person to take into consideration a stagnation point flow caused by a stretched flat plate. Later, Khashi'ie et al.^4^ did research on the stagnation point flow approaching a deformable flat plate. Disc, wedge, and cylinder fluid flows have all been studied more thoroughly in recent years because of technological and industrial advancements.

Encapsulating nanoparticles smaller than 100 nm in ethylene glycol, oil, or water might turn these standard heat transfer fluids into nanofluids. Ethylene glycol, oil, and water are lower heat transfer fluids because they have little or non-existent thermal conductivity. When it comes to transferring heat from one medium to another, the fluid's thermal conductivity is an important factor to consider. Choi^5^ shown that adding a little amount of nanoparticles (less than 1 percent by volume) to ordinary heat transfer liquids might substantially double the fluids' thermal conductivity. In the presence of ceramic or metallic nanoparticles, nanofluids demonstrated a significant increase in thermal conductivity that could not be explained by traditional ideas^6^. The thermal conductivity of a nanofluid is influenced by a variety of factors, including particle size and shape, liquid layering at the surface, heat transport properties, and the effects of aggregation^7^.

Nanofluids enhanced thermal conductivity is now the subject of intense dispute in the scientific community. These nanofluids, on the other hand, have been shown to have a significant thermal conductivity because of their aggregations, according to several investigations^8^. Through the use of light scattering measurements, the micron-sized clusters formed by nanoparticles have been shown by Yang et al.^9^. Using nanoparticle volume fractions of 1%, thermal conductivity increases are followed by fast viscosity increases, according to Kwak and Kim^10^, which is suggestive of aggregation effects. Lee et al.^11^ reported that the surface charge of nanoparticles is critical to the thermal conductivity of nanofluids. Nanoparticle aggregation is influenced by several parameters, including the surface charge. The formation of aggregates is thus a critical issue in the use of any nanofluid in a hot environment.

Because of the difference in density, thermal stratification is one of the significant and naturally occurring processes that might take place. Molecules with a considerable density may gather at the base of the surface, while molecules with a low density may exchange places and rise to the top portion of the layer in most instances in which the density of a material shifts because of a change in temperature or when the characteristics of a homogeneous mixture are altered by the application of a different temperature. The issue of convective flow in a thermally stratified fluid is a significant one, and this form of flow occurs in a variety of situations, varying from corporate and industrial contexts to the climate and weather environments, among others. When there is a difference in water density, stratification occurs. There are several factors at play, such as the density of the water, which determines whether a certain amount of water will glide on top of another. All fluids enclosed by differently heated side walls have a thermal stratification. As a result, in recent decades, researchers in both theoretical and practical domains have begun to pay more attention to the thermodynamics of fluxes in thermally stratified fluid. Most thermal stratification is caused by variations in temperature or the presence of different densities of different fluids in a vertical stratified environment^12^. According to the boundary layer assumptions, using a vertical plate submerged in a thermally stratified porous medium, Thakar and Pop^13^ looked into free convection from this plate. Tiwari and Singh^14^ conducted research on natural convection in a thermally stratified fluid saturated porous media to understand how it occurs. “Mixed convective stagnation point flow of a thermally stratified hybrid ${\rm Cu}{-}{\rm Al}_{2}{\rm O}_{3}/water$ nanofluid over a permeable stretching/shrinking sheet” is studied by^15^.

There are two types of convection: free (natural) and forced. The phrase "mixed convection" refers to the mixture of these two types of convection. As mixed convections in nanofluid have extensive industrial implications, particularly in nanoscience, there have been several studies on nanofluid mixed convections in recent times. There has been a startling bit of research reported on the boundary layer problem in nanofluid mixed convection. Magnetic field, suction/injection, and nanoparticle volume friction were all studied by Tamim et al.^16^ to see how they affected mixed convection around a nanofluid's stagnation-point flow. “The mixed convection flow around the stagnation-point area over an exponentially stretching/shrinking sheet in a nanofluid”, on the other hand, was examined by Subhashini et al.^17^ for both suction and injection situations in a nanofluid. Researchers have shown a great deal of interest in the issue of mixed convection flow because of its prevalence in the industrial sectors. For instance, solar and nuclear collectors, heat pumps, and atmospheric boundary layer fluxes^18–20^ are all examples of places where mixed convection flow is used. For the modeling of nanofluid flow on a curved stretched sheet, Ref,^21^ investigated convective heat transfer and the KKL correlation. Many studies have investigated the properties of nanofluid, which is believed to improve thermal conductivity, by looking at a variety of aspects and situations^22–26^.

Magnetohydrodynamics (MHD) flow phenomena are essential and have gained a lot of attention because of their pragmatic potential in numerous industrial and engineering fields, such as fusion reactors; optical fiber filters; crystal growth; metal casting; optical grafting; and plastic sheet stretching. Lorentz forces are generated when electrical currents and magnetic fields interact with one another. As a result, MHD defines how a conducting fluid behaves in the presence of a magnetic field. Examining the effects of MHD flow on industrial and technical domains, such as MHD generators and sterilizing instruments, as well as magnetic resonance graphs and MHD flow meters in granular insulation, is critical. The final product's quality is highly dependent on the cooling pace, which is controlled by the magnetic field and electrically conducting fluids. N. Sandeep^27^. Nanoparticle form and magnetohydrodynamic stagnation-point flow in Carreau nanoliquid were studied side by side by Sandeep^27^. Sutter by fluid flow confined at a stagnation point with an angled magnetic field and thermal radiation influences was recently studied numerically, according to Sabir et al.^28^. According to a study by Sarada et al.^29^, non-Newtonian MHD nanofluid flow and heat transmission via a stretched thin sheet are affected by nonlinear and temperature jumps. Many studies have been conducted since then, including^30–37^ that include MHD considerations.

Because of the ever-increasing demands of the technology and manufacturing industries, a great number of academics and practitioners have focused their efforts on developing methods that improve the study of heat transfer features and boundary layer characteristics beyond the use of a stretching/shrinking sheet. Industry uses include glass blowing, plastic sheet extrusion, and drawing of plastic films as well as hot rolling. The rate of heat transfer between the surface that is stretching or shrinking, and the fluid flow has a significant impact on the quality of the final product in terms of the desired characteristics^38,39^.

In industrial activities where the qualities of the output are reliant on the variables of heat management, the heat source, in conjunction to the suction influence, contributes significantly to the management of heat transfer. To better understand how heat sources affect nanofluid boundary layer flow and thermal conduction, as well as various other features, several studies have been conducted in this area. Using numerical simulations, Rana and Bhargava^40^ looked into the influence of different kinds of nanoparticles on mixed convection flow of nanofluid down a vertical plate with a heat source and sink. It also talked by Pal and Mandal^41^ how microrotation and nanoparticles affect boundary layer flow when there isn't a uniform heat source or sink, suction, heat radiation, or magnetic fields. The impact of “heat generation/absorption and thermal radiation on the hydromagnetic three-dimensional mixed convection flow of nanofluid across a vertical stretching surface” was inspected in another article by Mondal et al.^42^.

However, a deeper glance at the literature on the above-stated subjects reveals several holes and flaws. Prior studies have not explored the mixed convective stagnation point flow of water-based nanofluid across a permeable stretching/shrinking sheet with the addition of heat generation/absorption, nanoparticle aggregation, thermal stratification, and MHD in their study framework, to our knowledge. Because of this information gap, the ultimate purpose of this study is to use the model of^43^ to undertake a numerical analysis of the nanoparticle aggregation impact with heat generation and absorption towards a permeable stretching/shrinking thermally stratified sheet on MHD flow in Al_2_O_3_–H_2_O and ${\rm Cu}{-}{\rm H}_{2}{\rm O}$ nanofluid. Nonlinear partial differential equations may be transformed into ordinary differential equations using correct similarity transformations on the original equations. Furthermore, the present research used the RKF with shooting method approach in the MATHEMATICA operating system to solve the issue. The results are also presented in the form of tables and other visual aids. This important contribution might assist in improving industrial output, particularly in the manufacturing and process industries. It is our intention to:

- Simulate the thermally stratified mixed convective stagnation point flow using a magnetic field, nanoparticle aggregation, suction, and separate heat sources across a stretching/shrinking sheet.
- Conduct a comparison study of the flow of the nanoliquid with and without the aggregation of the nanoparticles.
- Find out what each of the different ways that the factors affect the profiles.
- Flow profiles are looked at, and the results are compared to a limiting example from the literature.
- Find out how heat sources and thermal stratification interact with each other to affect the pace at which heat is transferred.

## Description of the problem

**Figure 1:**
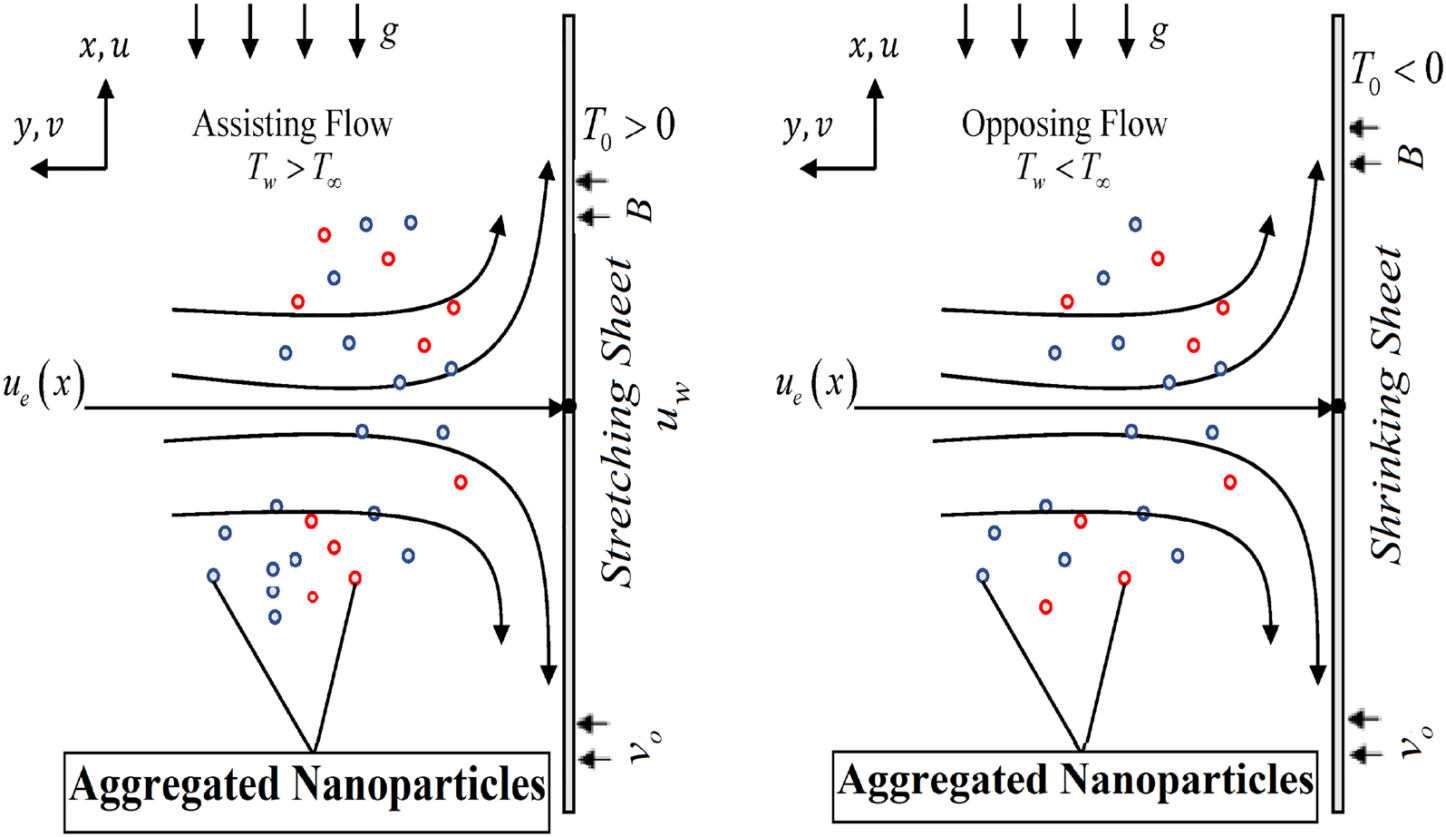
A visual representation of the physical model of mixed convective stagnation point flow across assisting and opposing surfaces.

A water-based nanofluid including two different types of nanoparticles (${\mathrm{Al}}_{2}{\mathrm{O}}_{3},\mathrm{Cu})$ with aggregation effects close to the stagnation area, passing through a permeable thermally stratified stretching sheet in the presence of a heat source with suction is depicted. Figure 1 displays the problem's physical arrangement in a schematic form. The free stream velocity is ${u}_{e}\left(x\right)=ax/L$, where $a$ is a constant and $L$ is the plate's characteristic length. ${u}_{w}\left(x\right)=bx/L,$ where $b$ is a constant. A variable magnetic field $B\left(x\right)={B}_{\circ }\frac{x}{L}$ is applied that is normal to the surface, ${B}_{\circ }$ being constant. In addition, a few assumptions about the physical model are investigated:

- Flow is laminar, steady, and incompressible.
- It does not include chemically activated species, such as joule heating, thermal radiation, viscous dissipation, or hall effect.
- The thermal equilibrium condition of the base fluid and nanoparticles is maintained.
- The nanoparticles have a spherical shape and are consistent in size.
- To facilitate fluid suction, the sheet is permeable.
- As a means of dealing with the issue of thermal stratification, it is believed that thermal buoyancy force is used^44–46^.
- The wall temperature is ${T}_{w}\left(x\right)={T}_{0}+B(x/L)$; $({T}_{w}>{T}_{\infty })$ designed to be used with a heated sheet (assisting flow) while $({T}_{w}<{T}_{\infty })$ in the case of a cooled sheet (opposing flow) (Fig. 1).
- The linear stratified ambient temperature may be written as ${T}_{\infty }={T}_{0}+A(x/L)$; ${T}_{0}$ represents the starting ambient temperature of nanofluid, $A$ represents a constant and $L$ represents the characteristic length of sheet.
- The modified Krieger–Dougherty and Maxwell–Bruggeman models are used to simulate the nanofluid's viscosity and thermal conductivity.

Considering all these above facts, the foremost flow equations are illustrated as^15,27,43^:

1 $$\frac{\partial \widetilde{u}}{\partial \mathrm{x}}+\frac{\partial \widetilde{v}}{\partial \mathrm{y}}=0,$$

2 $$\widetilde{u}\frac{\partial \widetilde{u}}{\partial \mathrm{x}}+\widetilde{v}\frac{\partial \widetilde{u}}{\partial \mathrm{y}}={\widetilde{u}}_{e}\frac{\partial {\widetilde{u}}_{e}}{\partial \mathrm{x}}+\frac{{\mu }_{nf}}{{\rho }_{nf}}\frac{{\partial }^{2}\widetilde{u}}{\partial {y}^{2}}+\frac{g{\left(\rho {\beta }_{T}\right)}_{nf}\left(T-{T}_{\infty }\right)}{{\rho }_{nf}}-\frac{{\sigma }_{nf}{B}^{2}}{{\rho }_{nf}}\left(u-{u}_{e}\right),$$

3 $$\widetilde{u}\frac{\partial \mathrm{T}}{\partial \mathrm{x}}+\widetilde{v}\frac{\partial \mathrm{T}}{\partial \mathrm{y}}=\frac{{k}_{nf}}{{\left(\rho {C}_{p}\right)}_{nf}}\frac{{\partial }^{2}T}{\partial {y}^{2}}+\frac{Q}{{\left(\rho {C}_{p}\right)}_{nf}}\left(T-{T}_{\infty }\right),$$

$\widetilde{u,}\widetilde{v}$ denotes the velocity component along the $x$ and $y$-axis, respectively. The $x$-axis measures beside the sheet, while the $y$-axis measures perpendicular to it. In addition, gravitational acceleration denoted by $g$, $T$ is the nanofluid temperature, ${\widetilde{u}}_{w}(x)$ corresponds to the stretching wall velocity, ${\widetilde{v}}_{w}\left(x\right)=-S\sqrt{\frac{a{\nu }_{f}}{L}}$ velocity of mass transport over permeable surface. It is possible to achieve the following boundaries:

4 $$\widetilde{u}={\widetilde{u}}_{w}\left(x\right),$$

5 $$\widetilde{v}={\widetilde{v}}_{w}\left(x\right), T={T}_{w}\left(x\right)\,\,\mathrm{ at\,\, y}=0,$$

6 $$\widetilde{u}\to {\widetilde{u}}_{e}\left(x\right), T\to {T}_{\infty },\,\,\mathrm{ as }\,\,y\to \infty$$

**Figure 2:**
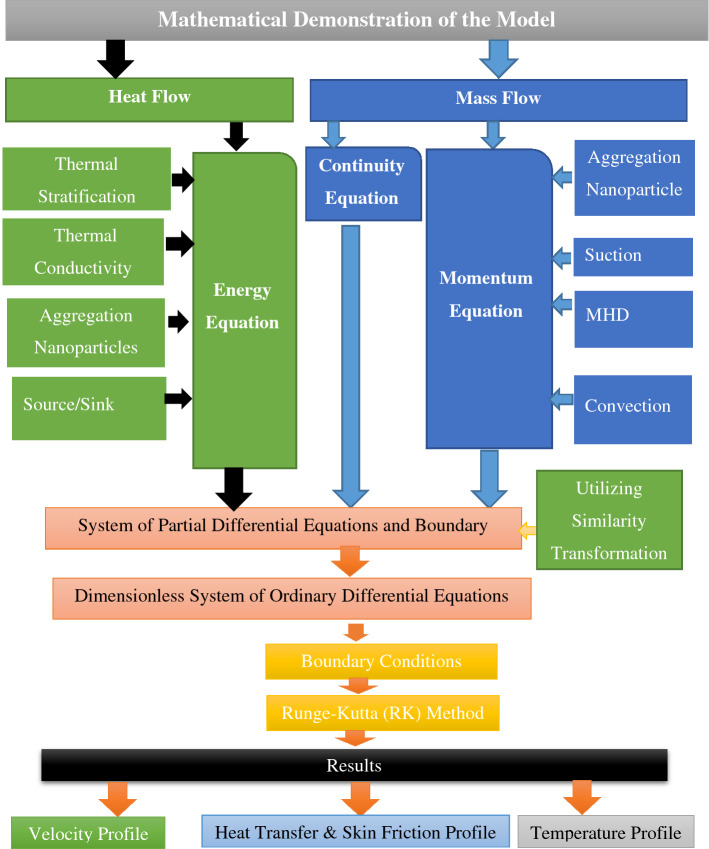
Model simulation.

Nanofluid properties such as dynamic viscosity ${\mu }_{nf}$, density ${\rho }_{nf}$, thermal expansion coefficient ${\left(\rho {\beta }_{T}\right)}_{nf}$, thermal conductivity ${k}_{nf}$, and heat capacitance ${\left(\rho {C}_{p}\right)}_{nf}$ are all mentioned here in. In the present work, the nanoparticles are alumina $({\rm Al}_{2}{\rm O}_{3})$ and copper Cu water on the other hand, serves as the primary fluid. Table 1 displays the thermophysical characteristics of the chosen nanoparticles and the base fluid. Table 2 summarize the thermophysical properties for nanofluids (see^43^). Model simulation is depicted in Fig. 2. The table below provides several thermos physical properties for aluminum oxide, copper, and water at $25^\circ C$ as the base fluid^15^:

**Table 1:** Thermophysical properties of a nanofluid and base fluid^15^.

Properties	${\rm Al}_{2}{\rm O}_{3}$	$\mathrm{Cu}$	${\mathrm{H}}_{2}\mathrm{O}$
$\rho ({\rm kg/m}^{3})$	$3970$	$8933$	$998.3$
$Cp ({\rm J/kg\,K})$	$765$	$385$	$4182$
$k ({\rm W/mK})$	$40$	$400$	$0.6130$
${\beta }_{T} \,({\rm K}^{-1})$	$0.85\times {10}^{-5}$	$1.67\times {10}^{-5}$	$21\times {10}^{-5}$
$\sigma \,\left({\rm S\,m}^{-1}\right)$	$35\times {10}^{6}$	$59.6\times {10}^{6}$	$5.5\times {10}^{-6}$

**Table 2:** Models of nano liquid with effective thermophysical properties (see^47–49^).

Effective property	Without aggregation	With aggregation
Density	$\frac{{\rho }_{nf}}{{\rho }_{f}}=\left(1-\phi \right)+\phi \frac{{\rho }_{S}}{{\rho }_{f}}$	$\frac{{\rho }_{nf}}{{\rho }_{f}}=\left(1-{\phi }_{a}\right)+{\phi }_{a}\frac{{\rho }_{S}}{{\rho }_{f}}$
Dynamic viscosity	$\frac{{\mu }_{nf}}{{\mu }_{f}}=\frac{1}{{\left(1-\phi \right)}^{2.5}}$	$\frac{{\mu }_{nf}}{{\mu }_{f}}={\left(1-\frac{{\phi }_{a}}{{\phi }_{m}}\right)}^{\left[\eta \right]{\phi }_{m}}$
Specific heat capacity	$\frac{{\left(\rho {C}_{p}\right)}_{{\varvec{n}}{\varvec{f}}}}{{\left(\rho {C}_{p}\right)}_{{\varvec{f}}}}=\left(1-\phi \right)+\phi \frac{{\left(\rho {C}_{p}\right)}_{{\varvec{S}}}}{{\left(\rho {C}_{p}\right)}_{{\varvec{f}}}}$	$\frac{{\left(\rho {C}_{p}\right)}_{{\varvec{n}}{\varvec{f}}}}{{\left(\rho {C}_{p}\right)}_{{\varvec{f}}}}=\left(1-{\phi }_{a}\right)+{\phi }_{a}\frac{{\left(\rho {C}_{p}\right)}_{{\varvec{S}}}}{{\left(\rho {C}_{p}\right)}_{{\varvec{f}}}}$
Thermal conductivity	$\frac{{k}_{nf}}{{k}_{f}}=\frac{\left({k}_{S}+2{k}_{f}\right)-2\phi \left({k}_{f}-{k}_{S}\right)}{\left({k}_{S}+2{k}_{f}\right)+\phi \left({k}_{f}-{k}_{S}\right)}$	$\frac{{k}_{nf}}{{k}_{f}}=\frac{\left({k}_{a}+2{k}_{f}\right)-2{\phi }_{a}\left({k}_{f}-{k}_{a}\right)}{\left({k}_{a}+2{k}_{f}\right)+{\phi }_{a}\left({k}_{f}-{k}_{a}\right)}$
Thermal expansion	${\left(\rho {\beta }_{T}\right)}_{nf}=\left(1-\phi \right){\left(\rho {\beta }_{T}\right)}_{f}+\phi {\left(\rho {\beta }_{T}\right)}_{s}$	${\left(\rho {\beta }_{T}\right)}_{nf}=\left(1-{\phi }_{a}\right){\left(\rho {\beta }_{T}\right)}_{f}+{\phi }_{a}{\left(\rho {\beta }_{T}\right)}_{s}$
Electrical conductivity	$\frac{{\sigma }_{nf}}{{\sigma }_{f}}=1+\frac{3\left(\frac{{\sigma }_{nf}}{{\sigma }_{f}}-1\right)\phi }{\left(\frac{{\sigma }_{nf}}{{\sigma }_{f}}+2\right)-\left(\frac{{\sigma }_{nf}}{{\sigma }_{f}}-1\right)\phi }$	$\frac{{\sigma }_{nf}}{{\sigma }_{f}}=1+\frac{3\left(\frac{{\sigma }_{nf}}{{\sigma }_{f}}-1\right){\phi }_{a}}{\left(\frac{{\sigma }_{nf}}{{\sigma }_{f}}+2\right)-\left(\frac{{\sigma }_{nf}}{{\sigma }_{f}}-1\right){\phi }_{a}}$

## Thermophysical properties

There are several variables to consider when calculating these equations. The nanofluid has a solid volume fraction $\phi$, ${\mu }_{nf}$ is dynamic viscosity characterizes the efficient nanofluid, and the base fluid density is ${\rho }_{f}$. ${\rho }_{nf}$ nanofluid density, $\left({\left({\rho C}_{p}\right)}_{nf}\right)$ is the heat capacity of nanofluids. In nanofluids, thermal conductivity is represented by ${k}_{nf}$, while in basic fluids it is represented by ${k}_{f}$.

To account for nanoparticle aggregation, the Krieger–Dougherty model was tweaked as indicated in Table 2. It is denoted by the symbol ${\phi }_{a}$, which stands for aggregate volume friction divides by the highest total packing fraction, which is provided by the formula $\left({\phi }_{a}=\phi {\left(\frac{{{\varvec{r}}}_{{\varvec{a}}}}{{{\varvec{r}}}_{{\varvec{p}}}}\right)}^{3-{\varvec{D}}}\right)$. Considering the spherical aggregation and diffusion-limited aggregation, it agrees with the experimental results of Alumina and copper nano liquids $D=1.8,\frac{{{\varvec{r}}}_{{\varvec{a}}}}{{{\varvec{r}}}_{{\varvec{p}}}}=3.34,{\phi }_{{\varvec{m}}}=0.605$ and $\left[\eta \right]=2.5$ (see^43^). The Brugman model was used in conjunction with the Maxwell model to create the aggregation model of thermal conductivity, which was subsequently modified. To determine the thermal conductivity of the aggregate $\left({k}_{a}\right)$ (^43^), use the following formula:

$$\frac{{k}_{a}}{{k}_{f}}=\frac{1}{4}\left\{\left(3{\phi }_{in}-1\right)\frac{{k}_{S}}{{k}_{f}}+\left(3\left(1-{\phi }_{in}\right)-1\right)+{\left[{\left(\left(3{\phi }_{in}-1\right)\frac{{k}_{S}}{{k}_{f}}+\left(3\left(1-{\phi }_{in}\right)-1\right)\right)}^{2}+8\frac{{k}_{S}}{{k}_{f}}\right]}^\frac{1}{2}\right\}.$$

Here, ${\phi }_{in}={\left(\frac{{{\varvec{r}}}_{{\varvec{a}}}}{{{\varvec{r}}}_{{\varvec{p}}}}\right)}^{{\varvec{D}}-3}.$ Eq. (7) fulfilled the continuity Eq. (1) as a result of applying the similarity transformation to the original data by (see^15^)

7 $$\widetilde{u}=\frac{ax}{L}{f}^{{\prime}}\left(\eta \right),\quad \widetilde{v}=-\sqrt{\frac{a{\upsilon }_{f}}{L}}f\left(\eta \right),\quad\theta \left(\eta \right)=\frac{T-{T}_{\infty }(x)}{{T}_{w}\left(x\right)-{T}_{0}},\quad\eta =\sqrt{\frac{a}{{\upsilon }_{f}L}}y.$$

In this case, prime denotes differentiation in reference to η. While ${\widetilde{v}}_{w}=-\sqrt{\frac{a{\nu }_{f}}{L}}S$. The following ordinary differential equations are drawn by including (1), (2), (5) and (6) in the steady-state equations and reduced boundary equations are

8 $${\mathrm{f}}^{{\prime\prime\prime}}+\frac{{\rho }_{nf}/{\rho }_{f}}{{\mu }_{nf}/{\mu }_{f}}\left[f{f}^{{\prime\prime}}-{f}^{{{\prime}}2}++\left(\frac{{\left(\rho {\beta }_{T}\right)}_{nf}/{\left(\rho {\beta }_{T}\right)}_{f}}{{\rho }_{nf}/{\rho }_{f}}\right)\lambda \theta +\frac{{\sigma }_{nf}/{\sigma }_{f}}{{\rho }_{nf}/{\rho }_{f}}M\left({1-f}^{{\prime}}\right)\right]=0,$$

9 $${\left(\frac{{k}_{nf}}{{k}_{f}}\right)}\uptheta^{{\prime\prime}}+\mathrm{Pr}\frac{{\left(\rho {C}_{p}\right)}_{{\varvec{n}}{\varvec{f}}}}{{\left(\rho {C}_{p}\right)}_{{\varvec{f}}}}\left[f{\uptheta }^{{{\prime}}}+\left\{{S-f}^{{\prime}}(\uptheta +\upgamma )\right\}\right]=0.$$

$$f\left( \eta \right)=\varepsilon , {f}^{{\prime}}\left(\eta \right)=c, \theta \left(\eta \right)=1-\upgamma $$ at $$\eta =0$$

10 $${f}^{{\prime}}\left(\eta \right)\to 1, \theta \left(\eta \right)\to 0\,\, as\,\, \eta \to \infty .$$

Among the parameters that occur in Eqs. (8)–(10) are the following:

i. $\lambda  = Gr/Re_{{x^{2} }}$ signifies the mixed convection parameter where $\lambda >0$ implies to the assisting flow,$\lambda <0$ denotes opposing flow, and $\lambda =0$ signifies pure forced convective flow. Further, $Gr=g{\left({\beta }_{T}\right)}_{f}\left({T}_{w}\left(x\right)-{T}_{\infty }\right){x}^{3}/{\nu }_{{f}^{2}}$ is the Grashof number and ${Re}_{x}=x{u}_{e}/{\nu }_{f}$ is the local Reynolds number.
ii. Prandtl number $Pr={\left({C}_{p}\mu \right)}_{f}/{k}_{f}$,
iii. Mass transpiration parameter $\varepsilon$ where $\varepsilon > 0$ denotes the suction parameter,
iv. $c=\frac{b}{a}$ is the stretching parameter.
v. $S=\frac{LQ}{{\left(\rho {C}_{p}\right)}_{nf}}$ is the source parameter.
vi. $M=\frac{\sigma {{B}^{2}}_{\circ }}{{\rho }_{nf}}$ is magnetic field.
vii. $\gamma =\frac{A}{B}$ is thermal stratification parameter.

Where $\upeta$ is the similarity variable, $f(\eta )$ is the dimensionless stream function, $\theta (\eta )$ is the dimensionless temperature and prime denotes the differentiation with respect to $\eta$.

### Physical quantities of interest

Local skin friction coefficient ${C}_{f}$ and local Nusselt number ${Nu}_{x}$ are

11 $${C}_{f}=\frac{{\tau }_{w}}{{\rho }_{f}{{u}^{2}}_{e}}\,\,\mathrm{ and }\,\,{Nu}_{x}=\frac{x{q}_{w}}{{k}_{f}\left({T}_{w}\left(x\right)-{T}_{\infty }\right)}$$

Shear stress and heat flow are expressed as ${\tau }_{w}$ and ${q}_{w}$, respectively and having following form:

$${\tau }_{w}={\mu }_{nf}{\left(\frac{\partial u}{\partial y}\right)}_{y=0},\quad {q}_{w}=-{k}_{nf}{\left(\frac{\partial T}{\partial y}\right)}_{y=0}$$

The lowered skin friction coefficient and local Nusselt number (heat transfer rate) of the nanofluid may be calculated using Eqs. (8) and (13), which represent shear stress and surface heat flux.

$${{Re}_{x}}^{1/2}{C}_{f}=\frac{{\mu }_{nf}}{{\mu }_{f}}{f}^{{\prime\prime}}\left(0\right), {{Re}_{x}}^{-1/2}{Nu}_{x}=-\left(\frac{{k}_{nf}}{{k}_{f}}\right){\theta }^{{\prime}}\left(0\right),$$

Provided that $${Re}_{x}=\frac{{u}_{e}}{{\nu }_{f}}.$$

## Numerical procedure

**Figure 3:**
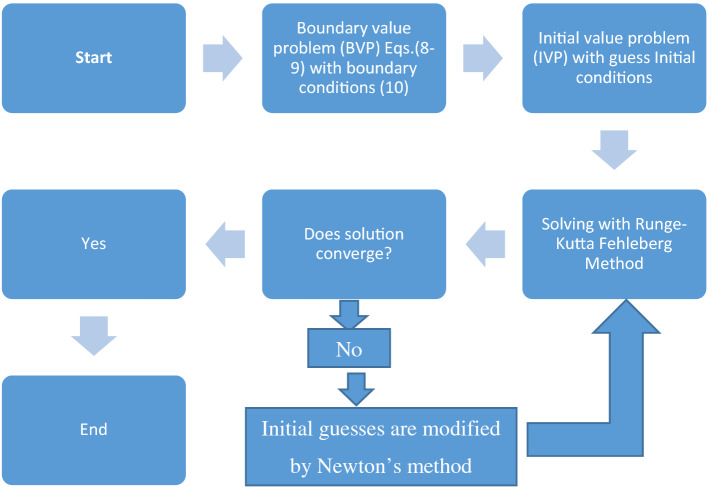
Schematic diagram for shooting method.

The Runge–Kutta–Fehlberg (RKF) along shooting technique is used to solve numerically the scheme of linked nonlinear differential Eqs. (8) and (9), together with the boundary conditions (10). Shooting approach is used to break down the system into a bunch of initial value issues, and the RKF method is used to find the solution. The step size $\Delta \eta =0.001$ is used to attain the numerical solution with ${\eta }_{max}=15,$ and a precision to the fifth decimal place as the standard of convergence. Schematic diagram for shooting method is shown in Fig. 3.

It is important to choose the starting approximations below to fulfil the convergence and boundary constraints (10).

12 $${\mathrm{m}}_{1}=f,\quad {\mathrm{m}}_{2}={f}^{{\prime}},\quad {\mathrm{m}}_{3}={f}^{{\prime\prime}},\quad {\mathrm{n}}_{1}=\theta ,{\mathrm{n}}_{2}={\theta }^{{\prime}},\quad  {\mathrm{n}}_{3}={\theta }^{{\prime\prime}}$$

using the Eq. (12)

13 $${{\mathrm{m}}^{{\prime}}}_{1}={f}^{{\prime}},\quad {{\mathrm{m}}^{{\prime}}}_{2}={f}^{{\prime\prime}},\quad {{\mathrm{m}}^{{\prime}}}_{3}={f}^{{\prime\prime\prime}},\quad {{\mathrm{n}}^{{\prime}}}_{1}={\theta }^{{\prime}},\quad {{\mathrm{n}}^{{\prime}}}_{2}=\theta {^{\prime\prime}}={\mathrm{n}}_{3},$$

we were able to get the following results from Eqs. (12) and (13).

14 $${{\mathrm{m}}^{{\prime}}}_{1}={\mathrm{m}}_{2},\quad {m}_{2}={m}_{3},\quad {m}_{3}={f}^{{\prime\prime\prime}},\quad {{\mathrm{n}}^{{\prime}}}_{1}={\mathrm{n}}_{2},\quad {{\mathrm{n}}^{{\prime}}}_{2}={\mathrm{n}}_{3}={\theta }^{{\prime\prime}},$$

When we arrange Eqs. (8–10) as bellows, we get the values ${f}^{{\prime\prime\prime}}$ and ${\theta }^{{\prime\prime}}$ that appear in Eq. (14):

15 $${m}_{3}=-\frac{{\rho }_{nf}/{\rho }_{f}}{{\mu }_{nf}/{\mu }_{f}}\left[{\mathrm{m}}_{1}{\mathrm{m}}_{3}-{{\mathrm{m}}_{2}}^{2}+1+\left(\frac{{\left(\rho {\beta }_{T}\right)}_{nf}/{\left(\rho {\beta }_{T}\right)}_{f}}{{\rho }_{nf}/{\rho }_{f}}\right)\lambda {\mathrm{n}}_{1}+M\left(1-{\mathrm{m}}_{2}\right)\right],$$

16 $${\mathrm{n}}_{3}=-\frac{1}{\left(\frac{{k}_{nf}}{{k}_{f}}\right)}\left[\mathrm{Pr}\frac{{\left(\rho {C}_{p}\right)}_{nf}}{{\left(\rho {C}_{p}\right)}_{f}}\left({\mathrm{m}}_{1}{\mathrm{n}}_{2}-{\mathrm{m}}_{2}{\mathrm{n}}_{1}+{\mathrm{Sn}}_{1}\right)\right].$$

17 $${\mathrm{m}}_{1}=\varepsilon , {\mathrm{m}}_{2}=\gamma , {\mathrm{n}}_{1}= 1, {\mathrm{m}}_{2} \to 1,{\mathrm{n}}_{1} \to 0.$$

Note that for all numerical simulation and graphical analysis a computational software Mathematica is used.

## Results and discussion

The numerical findings provided in Figs. 4, 5, 6, 7, 8, 9, 10, 11, 12, 13, 14, 15, 16, 17, 18, 19, 20, 21, 22, 23, 24, 25, 26, 27, 28, 29, 30, 31, 32, 33, 34, 35, 36, 37, 38, 39, 40, 41, 42, 43, 44, 45, 46, 47, 48, 49, 50, 51, 52 and 53 for various values of $c,\varepsilon ,M,\lambda ,\phi ,\gamma$ and $S$ are used to investigate the key aspects of flow and heat transmission. Extensive data on the thermophysical parameters of the fluids and nanoparticles $({\rm Al}_{2}{\rm O}_{3},Cu)$ employed in this investigation may be found in Table 1. Notably, except for comparisons with the preceding instance, the Prandtl number $Pr$ of the base fluid (water) is kept constant at 6.2. According to Tables 3 and 4, we compared our findings with those of Najiyah Safwa Khashi et al.^15^ and Rostami et al.^50^ for a variety of values of $Pr$, and found that they were in great agreement with our results. Therefore, we're confident in the precision of the current numerical technique. In the analysis, both with and without aggregation effects are used for Al_2_O_3_–H_2_O and ${\rm Cu}{-}{\rm H}_{2}{\rm O}$ respectively. Control factors are shown in tables and figures as they are modified. The far-field boundary conditions (10) dictate the values used here. The alumina and copper volume fractions are chosen between 0.0 percent and 4 percent. If the concentration of nanoparticles in the nanofluid is larger than 5% to 6%, the fluid may exhibit a non-Newtonian fluid behavior. Others are selected based on the major sources and potential solutions in an opposing flow, such as $-2.0\le \lambda \le 2.0$ (mixed convection), $0\le \varepsilon \le 2.0$ (suction), $0\le c\le 1.0$ (stretching), $0\le M\le 1.0$ (magnetic field), $0.01\le \gamma \le 0.6$(stratification), and $0\le S\le 1.0$ (source).

**Figure 4:**
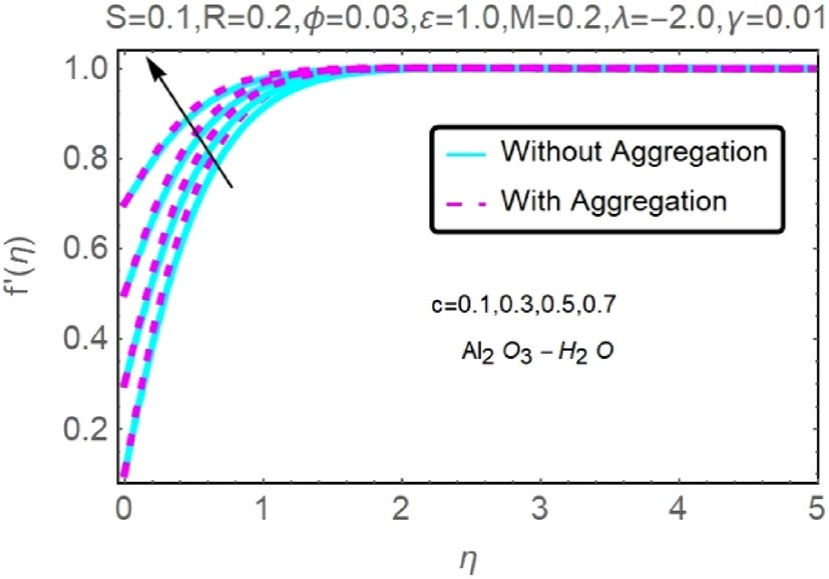
Fluctuation in $f{^{\prime}}(\eta )$ with varying estimates of $c$.

**Figure 5:**
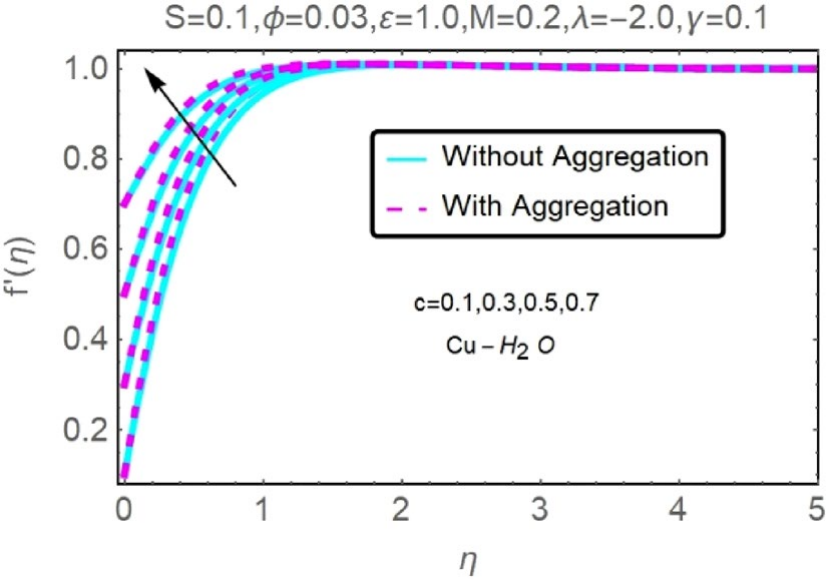
Fluctuation in $f{^{\prime}}(\eta )$ with varying estimates of $c$.

**Figure 6:**
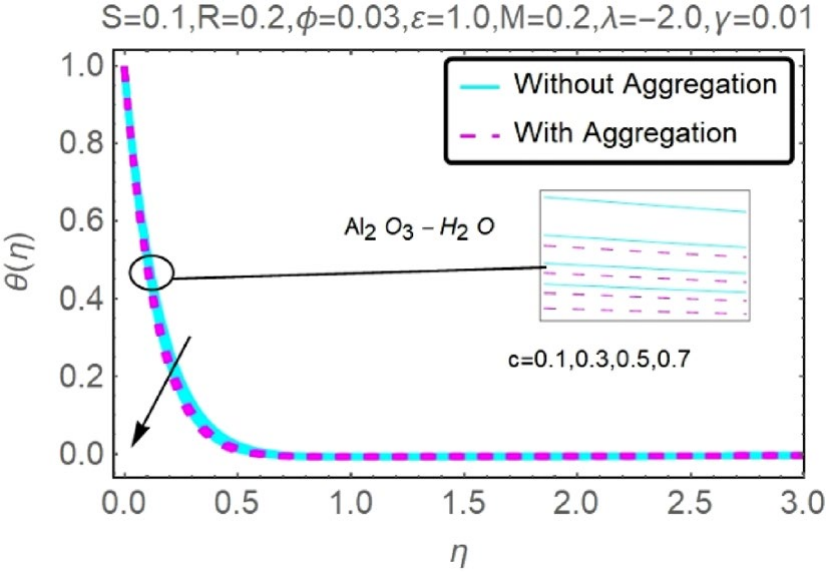
Fluctuation in $\theta (\eta )$ with varying estimates of $c$.

**Figure 7:**
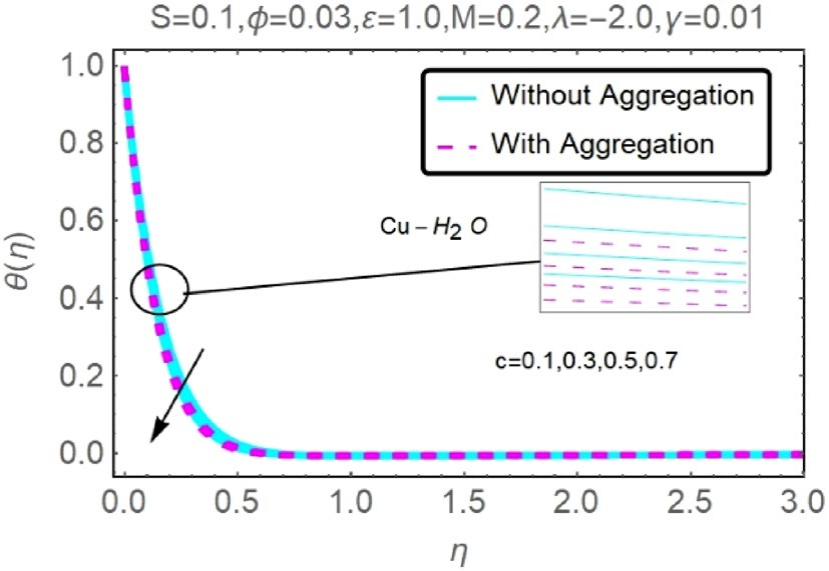
Fluctuation in $\theta (\eta )$ with varying estimates of $c$.

**Figure 8:**
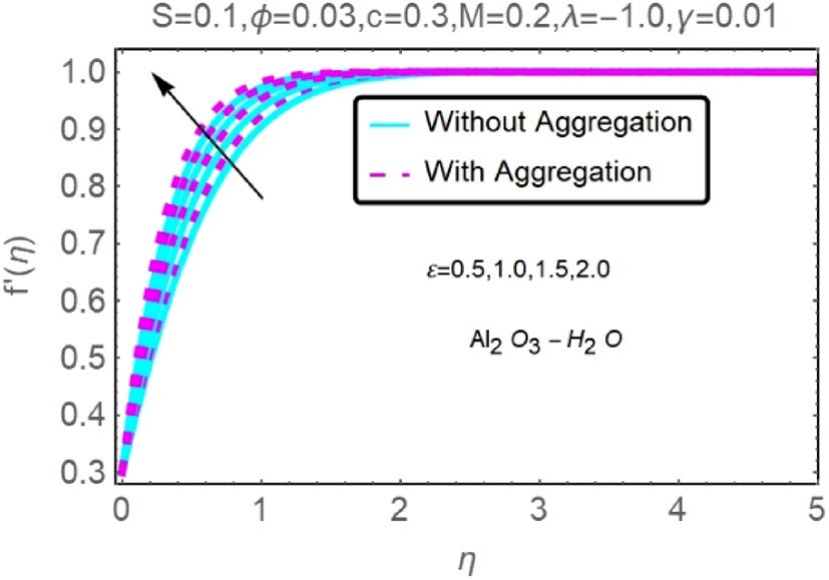
Fluctuation in $f{^{\prime}}(\eta )$ with varying estimates of $\varepsilon$.

**Figure 9:**
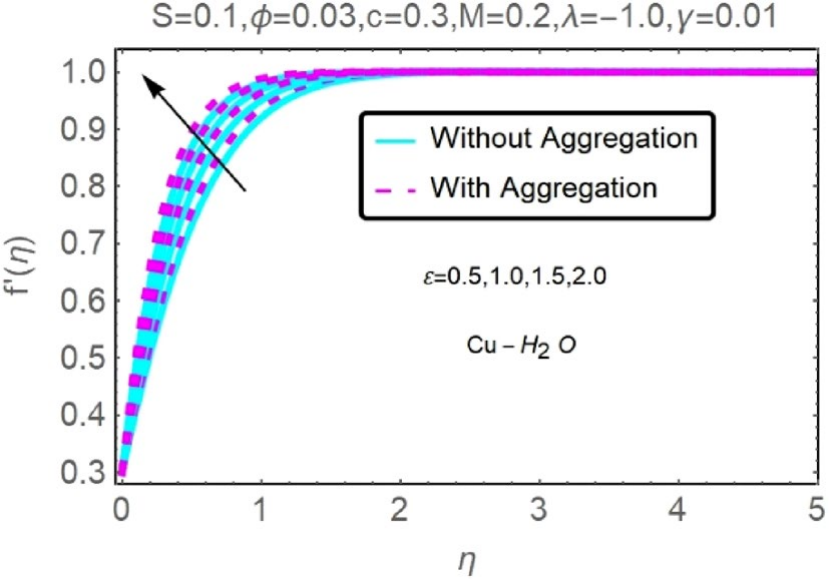
Fluctuation in $f{^{\prime}}(\eta )$ with varying estimates of $\varepsilon$.

**Figure 10:**
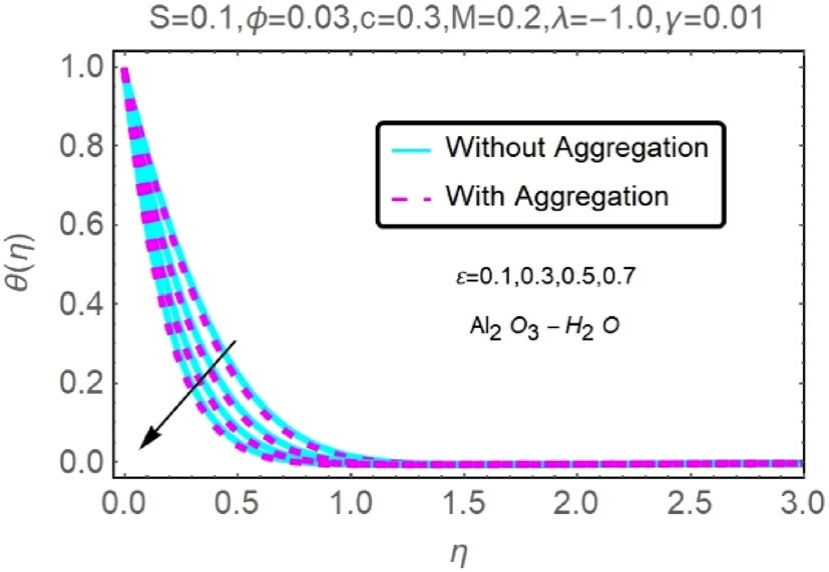
Fluctuation in $\theta (\eta )$ with varying estimates of $\varepsilon$.

**Figure 11:**
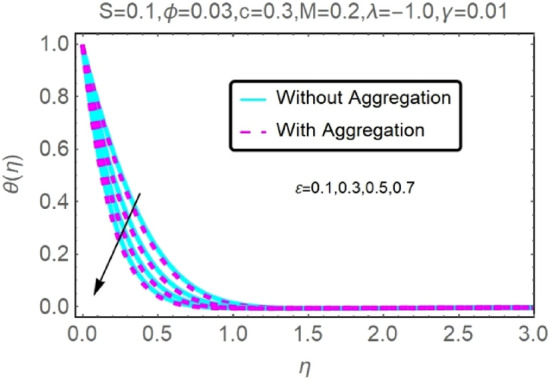
Fluctuation in $\theta (\eta )$ with varying estimates of $\varepsilon$.

**Figure 12:**
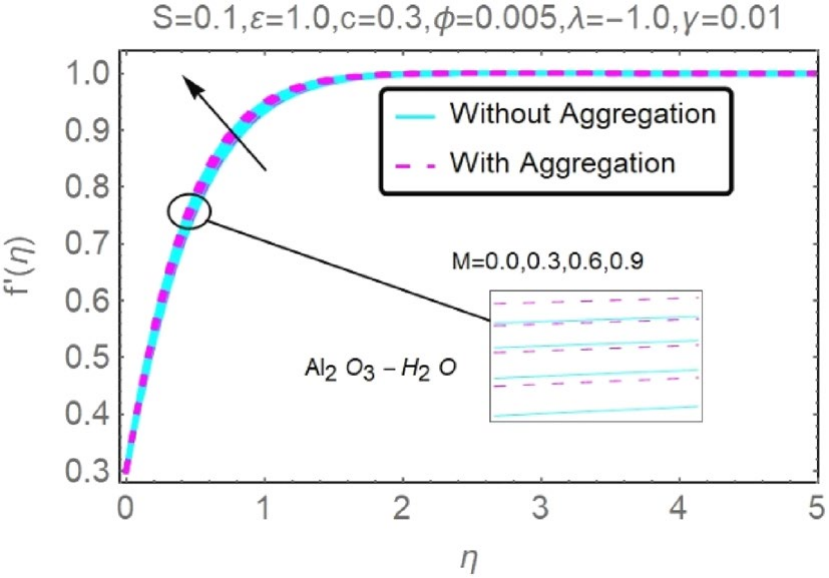
Fluctuation in $f{^{\prime}}(\eta )$ with varying estimates of $M$.

**Figure 13:**
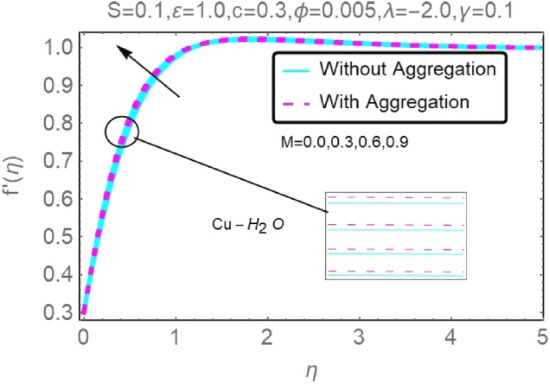
Fluctuation in $f{^{\prime}}(\eta )$ with varying estimates of $M$.

**Figure 14:**
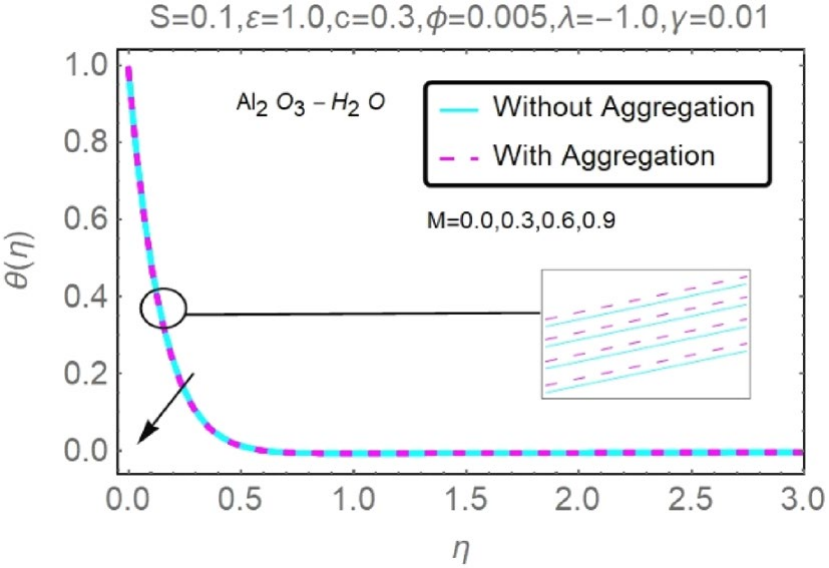
Fluctuation in $\theta (\eta )$ with varying estimates of $M$.

**Figure 15:**
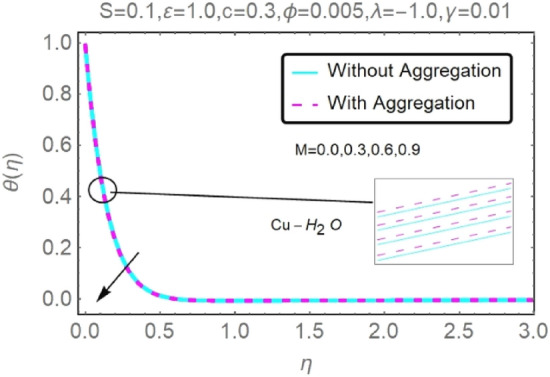
Fluctuation in $\theta (\eta )$ with varying estimates of $M$.

**Figure 16:**
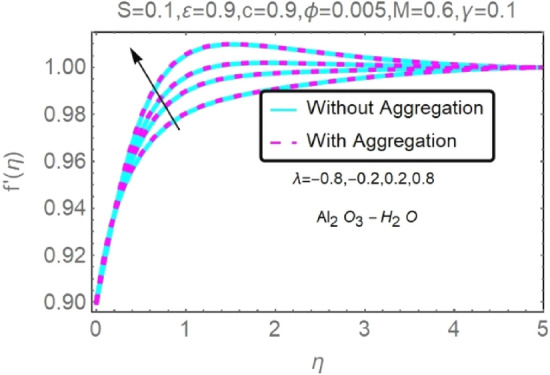
Fluctuation in $f{^{\prime}}(\eta )$ with varying estimates of $\lambda$.

**Figure 17:**
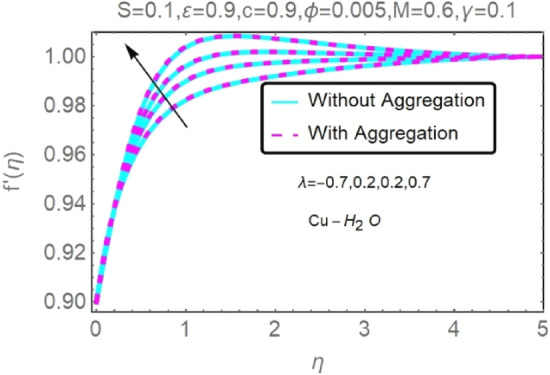
Fluctuation in $f{^{\prime}}(\eta )$ with varying estimates of $\lambda$.

**Figure 18:**
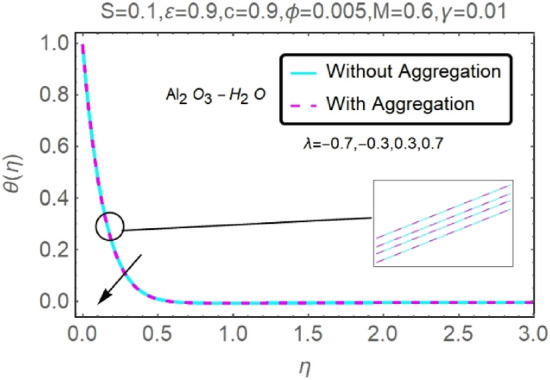
Fluctuation in $\theta (\eta )$ with varying estimates of $\lambda$.

**Figure 19:**
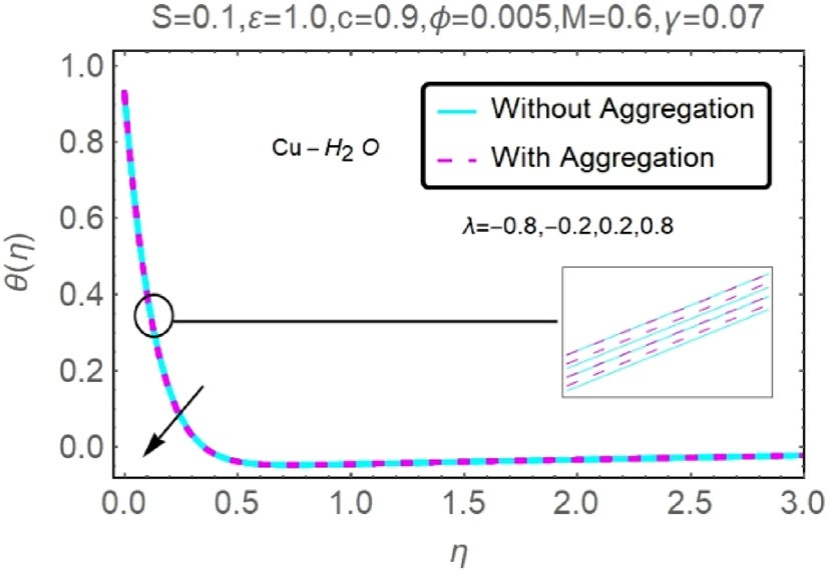
Fluctuation in $\theta (\eta )$ with varying estimates of $\lambda$.

**Figure 20:**
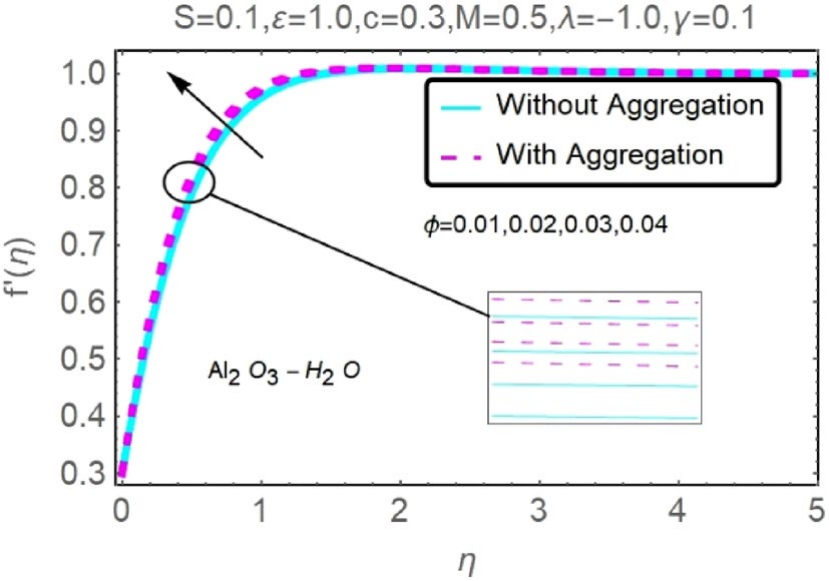
Fluctuation in $f{^{\prime}}(\eta )$ with varying estimates of $\phi$.

**Figure 21:**
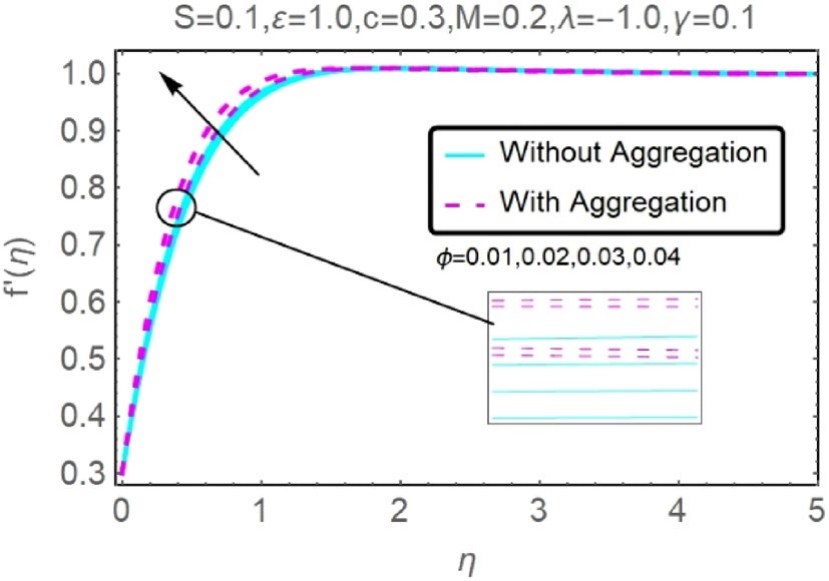
Fluctuation in $f{^{\prime}}(\eta )$ with varying estimates of $\phi$.

**Figure 22:**
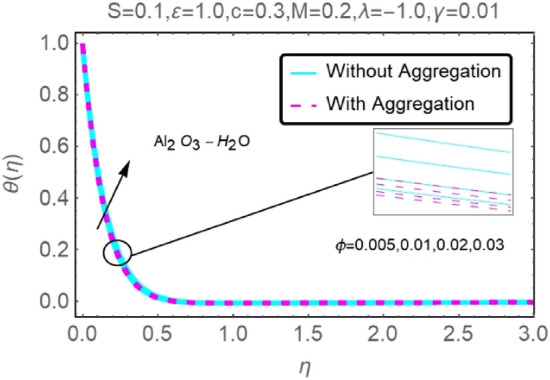
Fluctuation in $\theta (\eta )$ with varying estimates of $\phi$.

**Figure 23:**
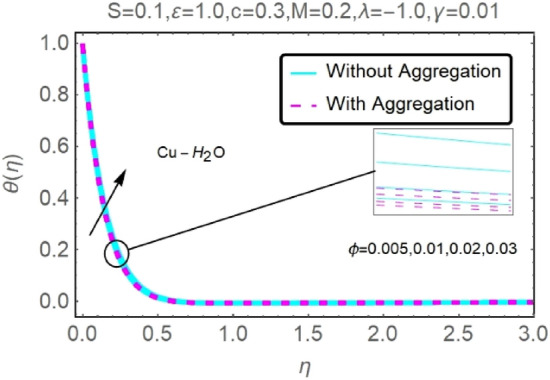
Fluctuation in $\theta (\eta )$ with varying estimates of $\phi$.

**Figure 24:**
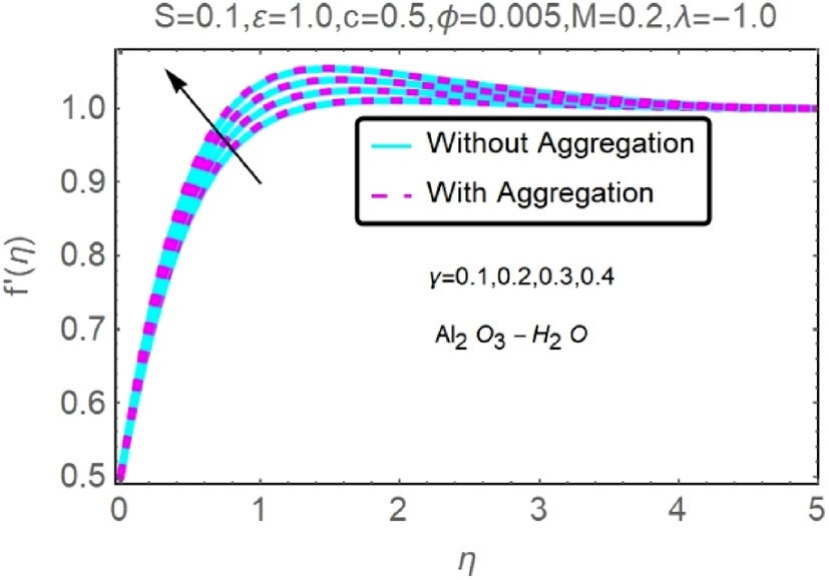
Fluctuation in $f{^{\prime}}(\eta )$ with varying estimates of $\gamma$.

**Figure 25:**
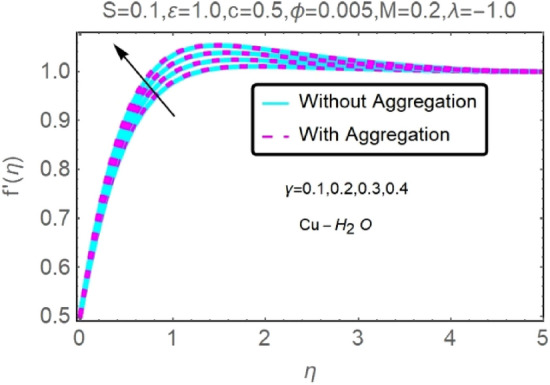
Fluctuation in $f{^{\prime}}(\eta )$ with varying estimates of $\gamma$.

**Figure 26:**
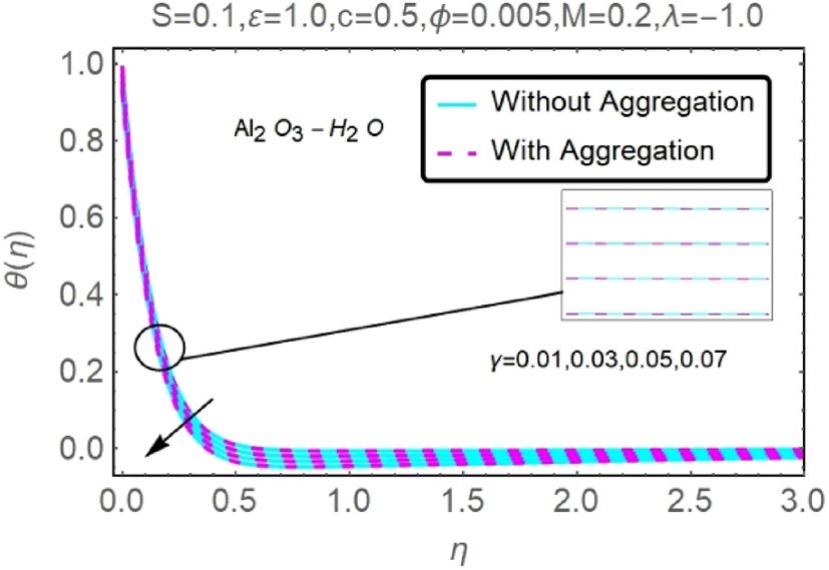
Fluctuation in $\theta (\eta )$ with varying estimates of $\gamma$.

**Figure 27:**
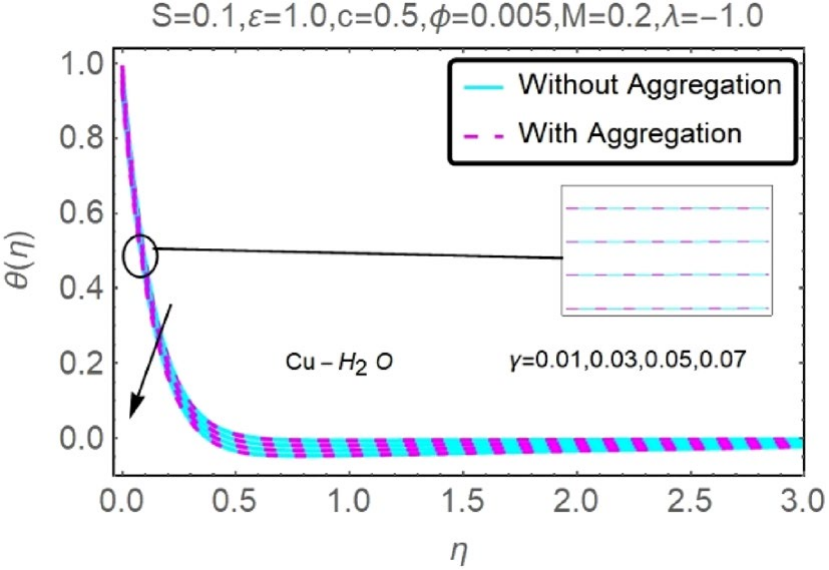
Fluctuation in $\theta (\eta )$ with varying estimates of $\gamma$.

**Figure 28:**
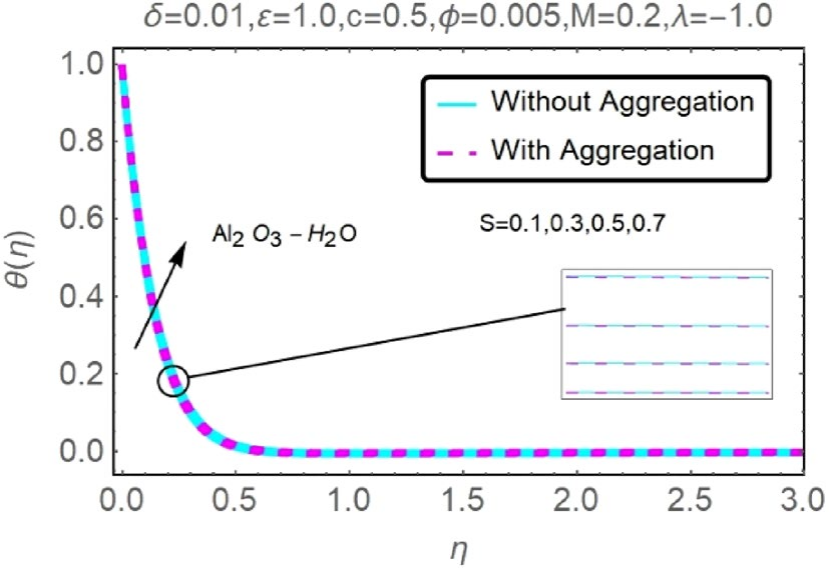
Fluctuation in $\theta (\eta )$ with varying estimates of $S$.

**Figure 29:**
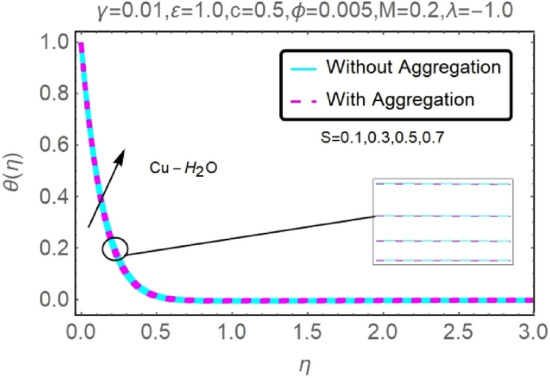
Fluctuation in $\theta (\eta )$ with varying estimates of $S$.

**Figure 30:**
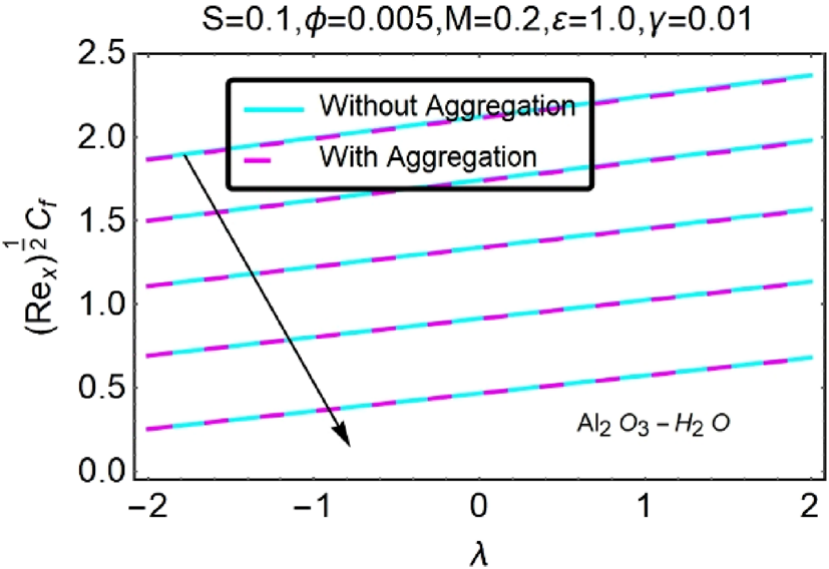
Variation in $({{Re}_{x}}^{1/2}{C}_{f})$ against $\lambda$ and $c$.

**Figure 31:**
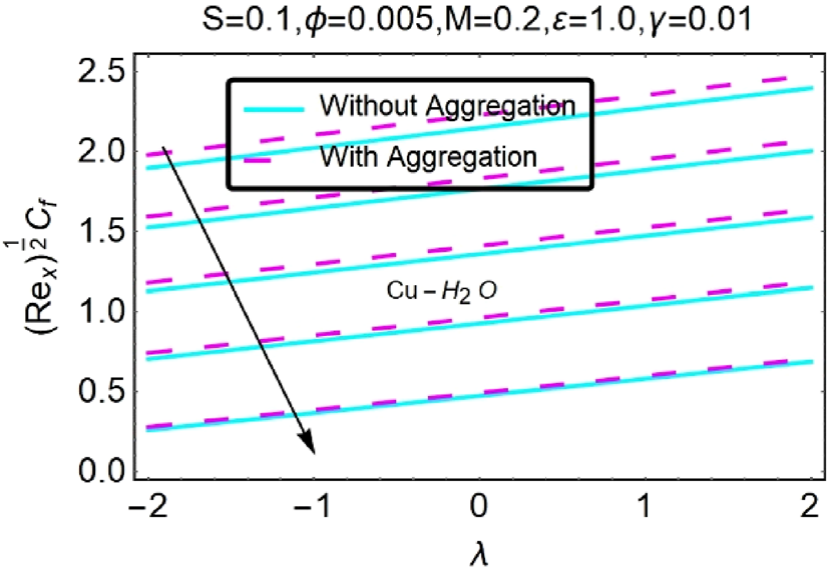
Variation in $({{Re}_{x}}^{1/2}{C}_{f})$ against $\lambda$ and $c$.

**Figure 32:**
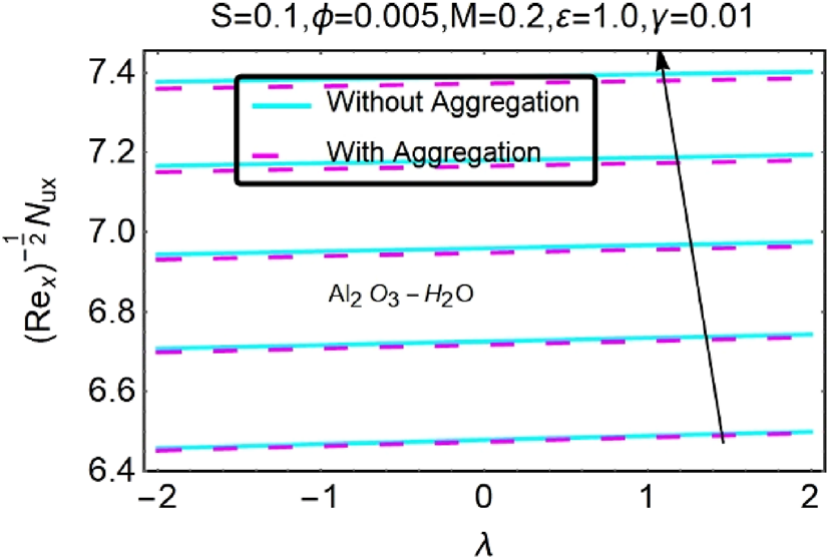
Variation in $\left({{Re}_{x}}^{-1/2}{Nu}_{x}\right)$ with various $\lambda$ and $c$.

**Figure 33:**
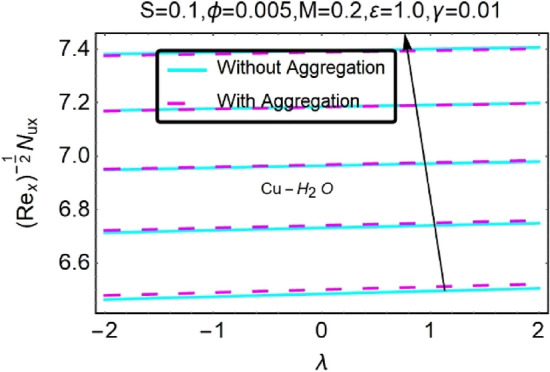
Variation in $\left({{Re}_{x}}^{-1/2}{Nu}_{x}\right)$ with various $\lambda$ and $c$.

**Figure 34:**
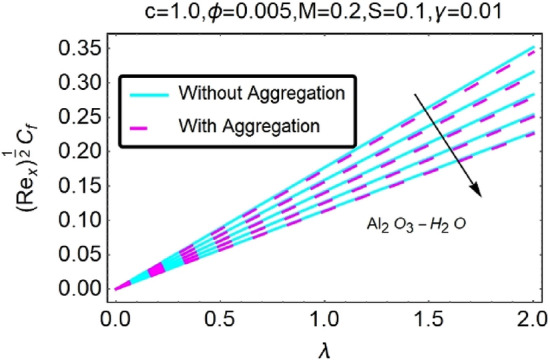
Variation in $({{Re}_{x}}^{1/2}{C}_{f})$ against $\lambda$ and $\varepsilon$.

**Figure 35:**
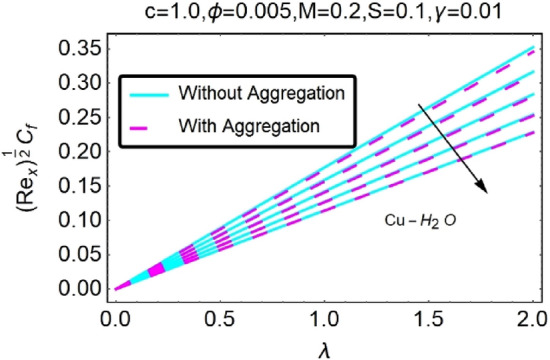
Variation in $({{Re}_{x}}^{1/2}{C}_{f})$ against $\lambda$ and $\varepsilon$.

**Figure 36:**
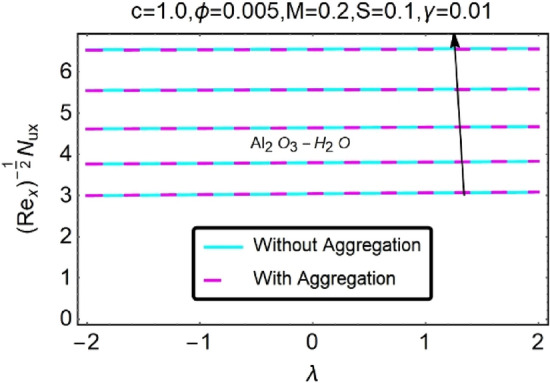
Variation in $\left({{Re}_{x}}^{-1/2}{Nu}_{x}\right)$ with various $\lambda$ and $\varepsilon$.

**Figure 37:**
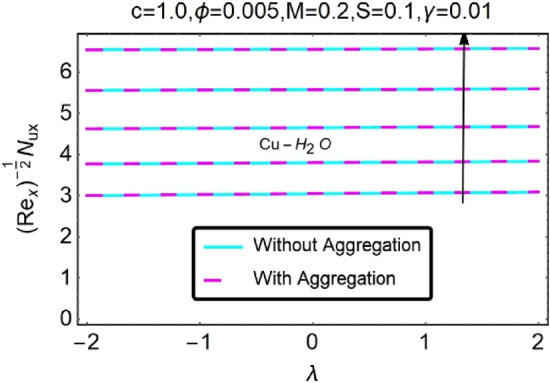
Variation in $\left({{Re}_{x}}^{-1/2}{Nu}_{x}\right)$ with various $\lambda$ and $\varepsilon$.

**Figure 38:**
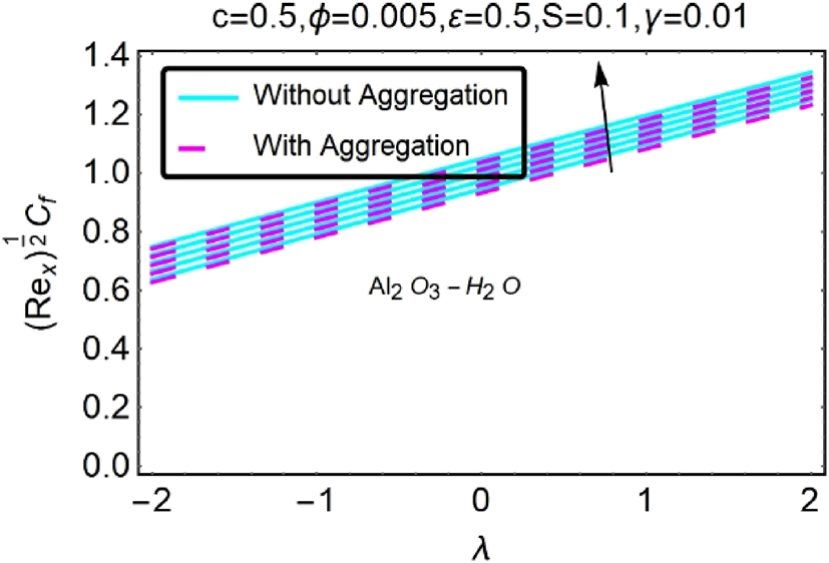
Variation in $({{Re}_{x}}^{1/2}{C}_{f})$ against $\lambda$ and $M$.

**Figure 39:**
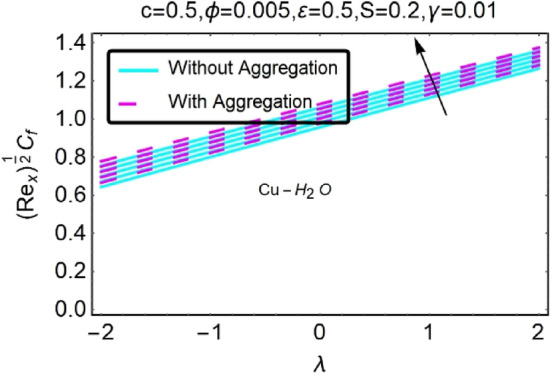
Variation in $({{Re}_{x}}^{1/2}{C}_{f})$ against $\lambda$ and $M$.

**Figure 40:**
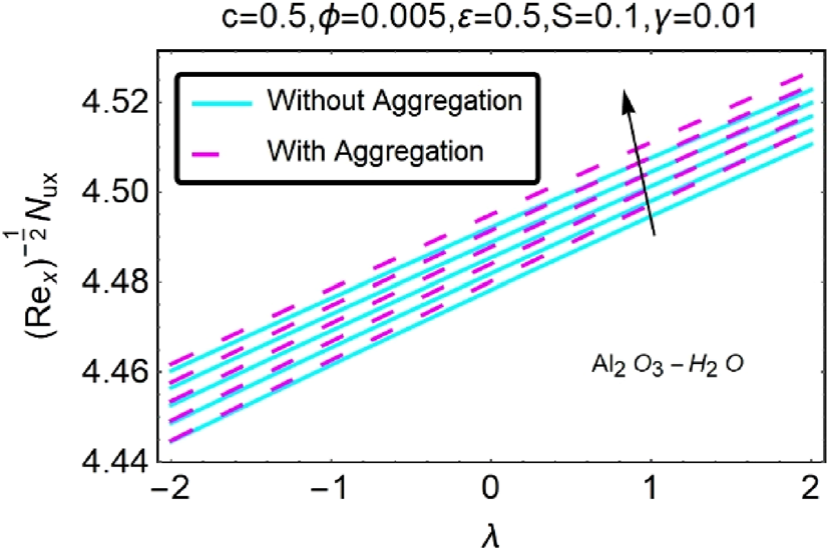
Variation in $\left({{Re}_{x}}^{-1/2}{Nu}_{x}\right)$ with various $\lambda$ and $M$.

**Figure 41:**
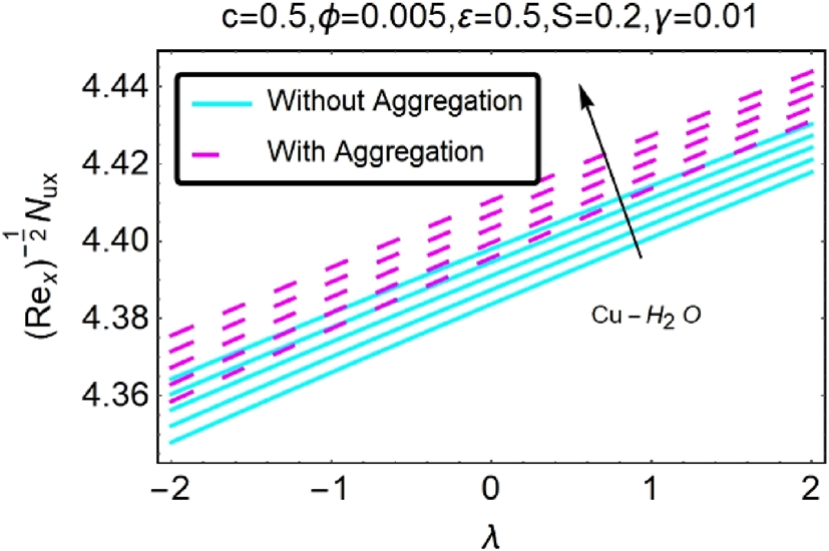
Variation in $\left({{Re}_{x}}^{-1/2}{Nu}_{x}\right)$ with various $\lambda$ and $M$.

**Figure 42:**
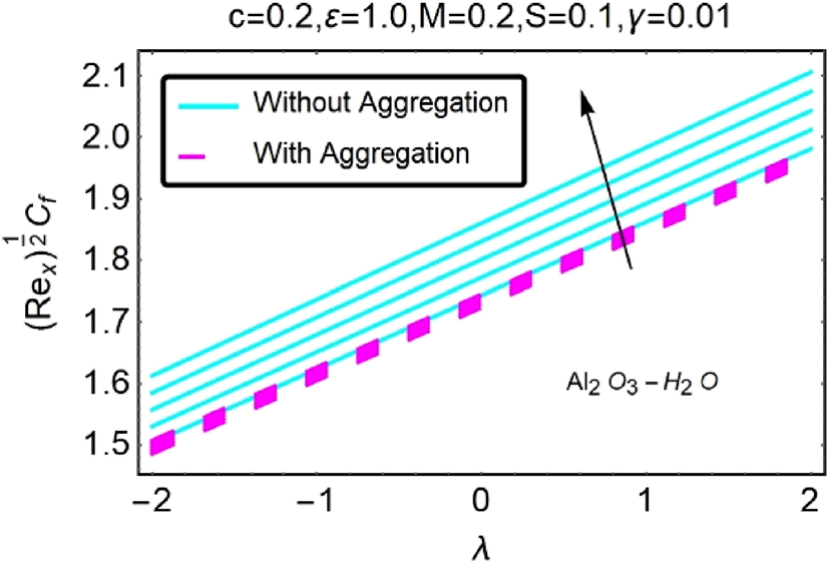
Variation in $({{Re}_{x}}^{1/2}{C}_{f})$ against $\lambda$ and $\phi$.

**Figure 43:**
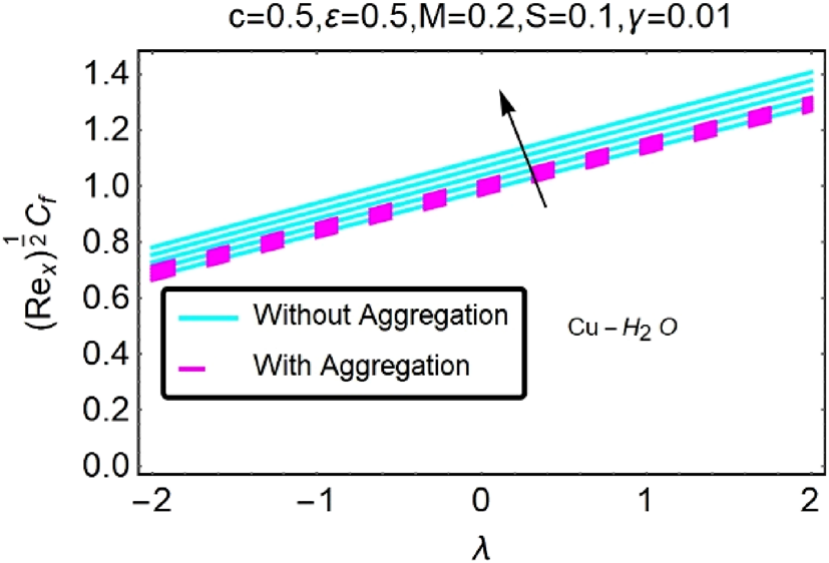
Variation in $({{Re}_{x}}^{1/2}{C}_{f})$ against $\lambda$ and $\phi$.

**Figure 44:**
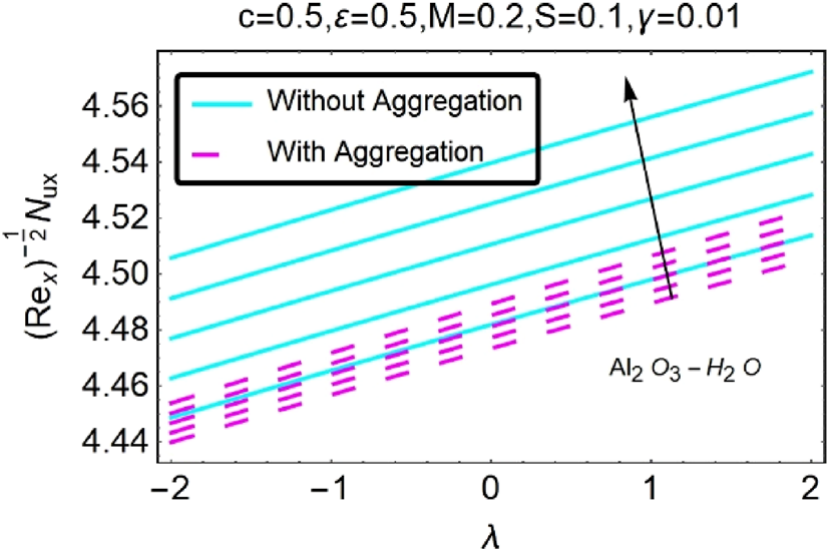
Variation in $\left({{Re}_{x}}^{-1/2}{Nu}_{x}\right)$ with various $\lambda$ and $\phi$.

**Figure 45:**
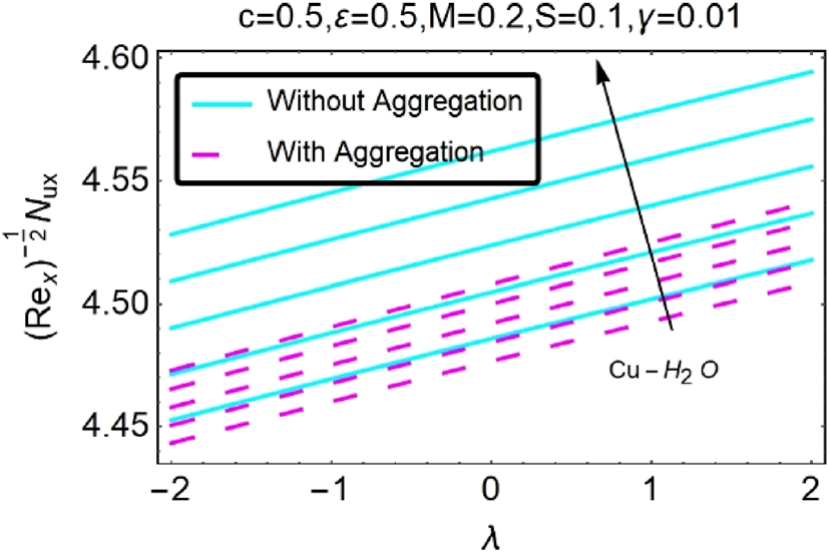
Variation in $\left({{Re}_{x}}^{-1/2}{Nu}_{x}\right)$ with various $\lambda$ and $\phi$.

**Figure 46:**
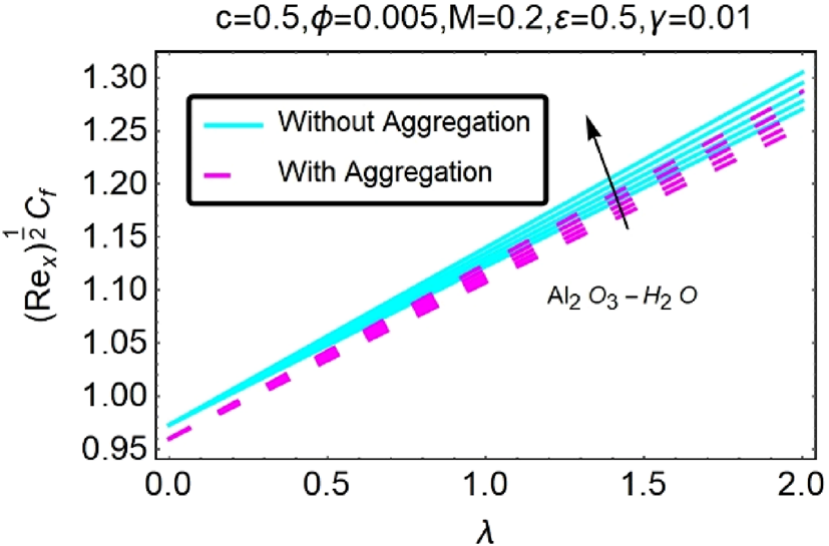
Variation in $({{Re}_{x}}^{1/2}{C}_{f})$ against $\lambda$ and $S$.

**Figure 47:**
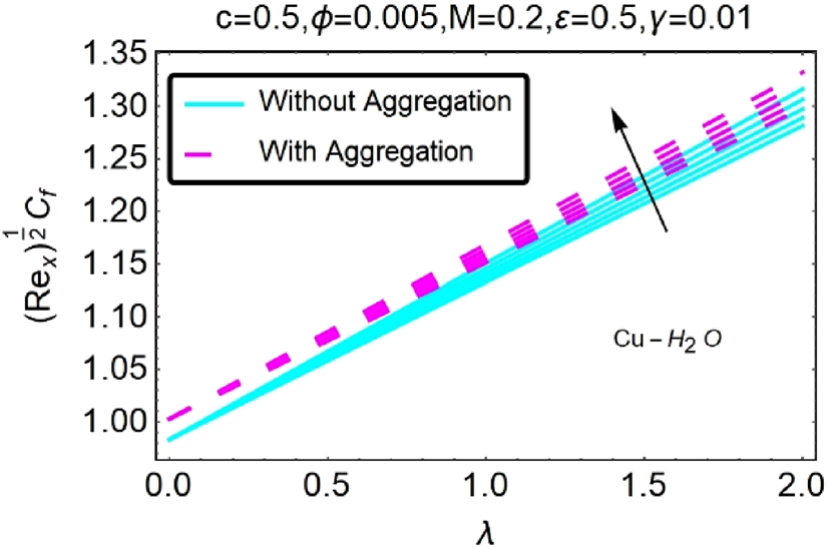
Variation in $({{Re}_{x}}^{1/2}{C}_{f})$ against $\lambda$ and $S$.

**Figure 48:**
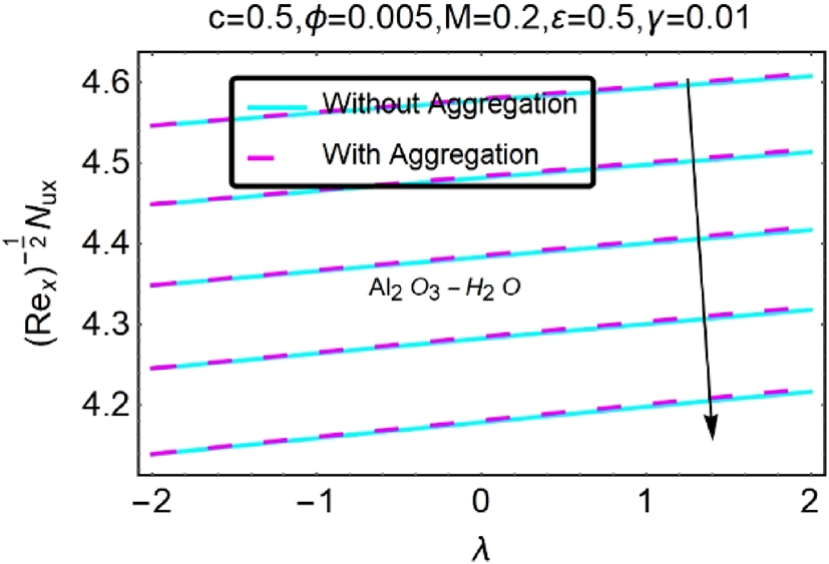
Variation in $\left({{Re}_{x}}^{-1/2}{Nu}_{x}\right)$ with various $\lambda$ and $S$.

**Figure 49:**
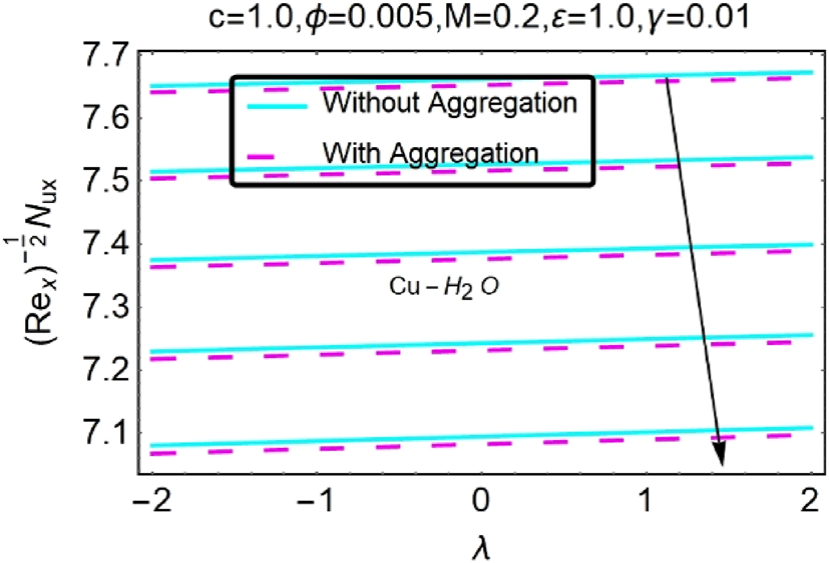
Variation in $\left({{Re}_{x}}^{-1/2}{Nu}_{x}\right)$ with various $\lambda$ and $S$.

**Figure 50:**
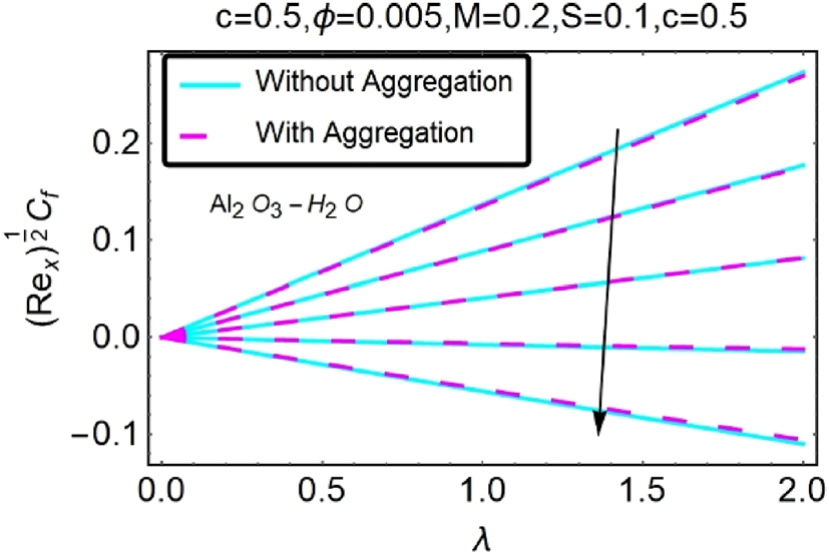
Variation in $({{Re}_{x}}^{1/2}{C}_{f})$ with various $\lambda$ and $\gamma$.

**Figure 51:**
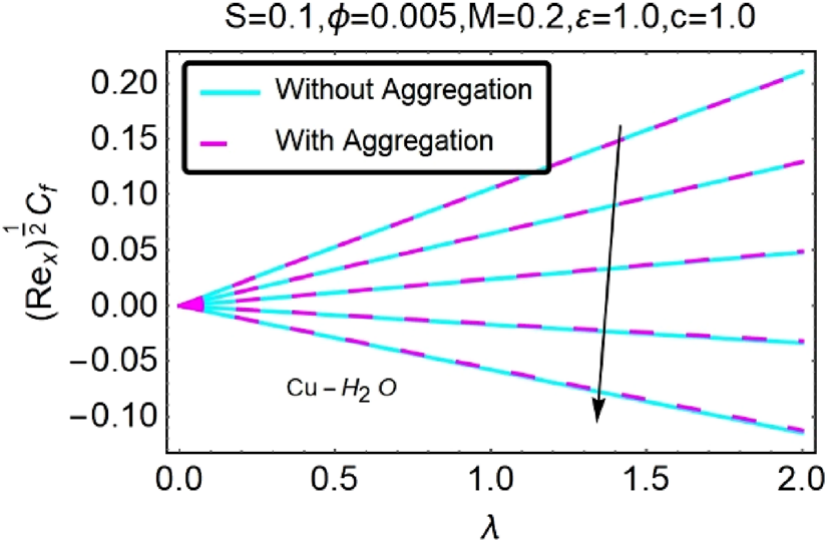
Variation in $({{Re}_{x}}^{1/2}{C}_{f})$ with various $\lambda$ and $\gamma$.

**Figure 52:**
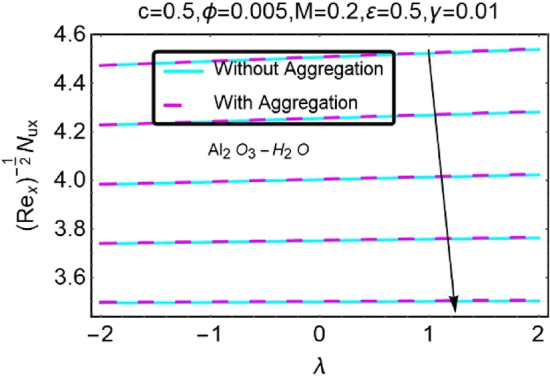
Variation in $\left({{Re}_{x}}^{-1/2}{Nu}_{x}\right)$ with various $\lambda$ and $\gamma$.

**Figure 53:**
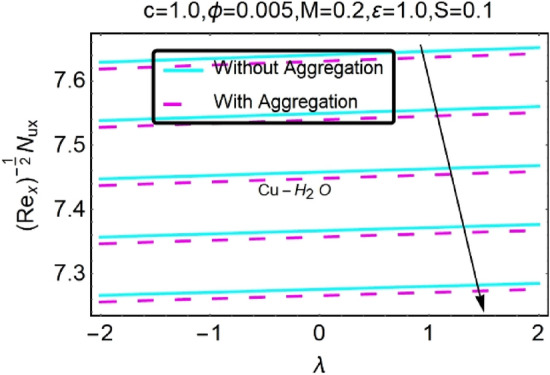
Variation in $\left({{Re}_{x}}^{-1/2}{Nu}_{x}\right)$ with various $\lambda$ and $\gamma$.

**Table 3:** ${f}^{{\prime\prime}}\left(0\right),$ $\phi ={\phi }_{a}=\gamma =\varepsilon =M=S=0,\lambda =1$ with respect to variety of $Pr.$

$Pr$	Present Results	Najiyah Safwa Khashi et al.^15^		Rostami et al.^50^	
	${\rm Al}_{2}{\rm O}_{3}/{\rm H}_{2}{\rm O}$&$Cu/{\rm H}_{2}{\rm O}$	First Sol	Second Sol	First Sol	Second Sol
0.7	1.72608	1.7063	1.2387	1.7063	1.2344
1	1.71179	1.6754	1.1332	1.6754	1.1296
7	1.57524	1.5179	0.5824	1.5179	0.5815

**Table 4:** $-\theta {^{\prime}}\left(0\right),$ when $\phi ={\phi }_{a}=\gamma =\varepsilon =M=S=0,\lambda =1$ $1$ with respect to variety of $Pr.$

$Pr$	Present Results	Najiyah Safwa Khashi et al.^15^		Rostami et al.^50^	
	${\rm Al}_{2}{\rm O}_{3}/{\rm H}_{2}{\rm O}$&$Cu/{\rm H}_{2}{\rm O}$	First Sol	Second Sol	First Sol	Second Sol
0.7	1.68677	0.7641	1.0226	0.7641	1.0235
1	1.69536	0.8708	1.1691	0.8708	1.1706
7	1.86382	1.7224	2.2192	1.7224	2.2203

It is possible to compare the results of Tables 3 and 4 to those of Najiyah Safwa Khashi et al.^15^ and Rostami et al.^50^ in the event that all physical factors except $\lambda =1$ are $0$. The bvp4c solver was used by Najiyah Safwa Khashi et al.^15^ and Rostami et al.^50^ to explore viscous fluid mixed convective stagnation point flow. A permeable flat plate ($c=0$) was examined in both existing literatures. Our findings are in excellent accord with those of Najiyah Safwa Khashi et al.^15^, as shown in Tables 3 and 4, as well as with Rostami et al.^50^. The researcher is thus comfortable in the usage of the current concept and procedure. Tables 3 and 4 provide all numerical values for the simplified case of a Newtonian fluid.

### Velocity ${\varvec{f}}{^{\prime}}({\varvec{\eta}})$ and temperature ${\varvec{\uptheta}}({\varvec{\upeta}})$ profiles

The velocity and temperature profiles of Al_2_O_3_–H_2_O and ${\rm Cu}{-}{\rm H}_{2}{\rm O}$ nanofluid values of $c,\varepsilon ,M,\lambda ,\phi ,\gamma$ and $S$ are elucidated in Figs. 4, 5, 6, 7, 8, 9, 10, 11, 12, 13, 14, 15, 16, 17, 18, 19, 20, 21, 22, 23, 24, 25, 26 and 27. To maintain the validity of the current solutions, the far-field boundary criteria must be met. The velocity profile $f{^{\prime}}(\eta )$ of the aggregation model is somewhat greater than that of the homogeneous model. Aggregation causes an increase in effective viscosity, that is why this happens.

The velocity and temperature profiles for two different types of nanofluids $({\rm Al}_{2}{\rm O}_{3}{-}{\rm H}_{2}{\rm O},{\rm Cu}{-}{\rm H}_{2}{\rm O})$ for without and with aggregation effects against stretching parameter $(c)$ are shown in Figs. 4, 5, 6 and 7. It should go without saying that increasing the value of $c$ leads the velocity field to grow. Increases in $c$ cause the thickness of momentum boundary layers to become thinner as time goes on, which has a physical consequence on the system. The stretching parameter $c$ affects the temperature. Figures 6 and 7 depict it. When a fluid is stretched further, its temperature drops. It can be observed from these figures that while stretching a sheet of ${\rm Cu}{-}{\rm H}_{2}{\rm O}$, the velocity distribution is greater, and the temperature distribution is lower than when stretching an Al_2_O_3_–H_2_O sheet. Nanofluids made of Al_2_O_3_–H_2_O and ${\rm Cu}{-}{\rm H}_{2}{\rm O}$ are shown to have similar effects, although the latter is more potent.

Figures 8, 9, 10 and 11 show the effects of suction $(\varepsilon )$ on the velocities and temperatures of Al_2_O_3_–H_2_O and ${\rm Cu}{-}{\rm H}_{2}{\rm O}$ nanofluids. As the suction parameter is raised, the velocities of Al_2_O_3_–H_2_O and ${\rm Cu}{-}{\rm H}_{2}{\rm O}$ both rises, but the temperature profile shows the reverse trend (Figs. 8, 9, 10 and 11). By reducing the thickness of the momentum barrier layer, suction improves flow at the plate surface.

According to Figs. 12, 13, 14 and 15, several values of $M$ are shown for velocity and temperature. To enhance fluid velocity, Because of the existence of a magnetic field through an electrically conducting fluid, the Lorentz force emerges across the fluid (see Figs. 12, 13). More nanoparticles are drawn to the surface by the Lorentz force ensuing in greater temperature near the permeable sheet. In addition, increasing the boundary layer's thickness may be achieved by an increase in fluid's magnetic influence, thereby reducing the convection mechanism dramatically over the permeable wall surface, as shown clearly in Figs. 14, 15.

Figures 16, 17, 18 and 19 demonstrate the influence of the buoyancy parameter $\lambda$ on the velocity and temperature profiles of Al_2_O_3_–H_2_O and ${\rm Cu}{-}{\rm H}_{2}{\rm O}$ nanofluid stretching sheets. When contrasting the impact of buoyancy on thermal and mass flow with the impact of inertia on an externally generated or free stream flow, the buoyancy coefficient ($\lambda$) is used. These graphs show that as $\lambda$ increases, the velocity profiles for stretched sheet grow, while the temperature profiles decrease.

Figures 20, 21, 22 and 23 show the varying velocities and temperatures of Al_2_O_3_–H_2_O and ${\rm Cu}{-}{\rm H}_{2}{\rm O}$ nanofluids in relation to $\phi$ of stretching sheet. These graphs show that as $\phi$ increases, velocity and temperature spectra of a stretched sheet follow the same pattern. Increases in velocity and temperature are caused by the accretion of nanoparticle volumetric concentration, which depletes more energy.

Figures 24 and 25 show that when the thermal stratification $\gamma$ value is raised, the velocity profile of both nanofluids rises. As the fluid's temperature rises via its thermal stratification, it becomes more difficult to maintain constant velocity. Thermal stratification parameter $\gamma$ effect on temperature profile for two types of nanofluids is shown in Figs. 26 and 27. As the stratification level increases, so does the temperature. This is because the surface area has a lower rate of heat transmission.

Heat source parameter $S$ has been displayed in Figs. 28 and 29 to examine the temperature profile fluctuation for various values. As was previously mentioned, the existence of the heat production parameter $S$ has the propensity to enhance the thermal state of the fluid and the temperature distribution close to the surface. The elevation of the fluid temperature prompts more induced flow to the surface, enabling the thermal boundary layer thickness to grow consequently, subsequently lowering the rate of heat transfer at the stretching/shrinking surface. Temperature profiles for Al_2_O_3_–H_2_O and ${\rm Cu}{-}{\rm H}_{2}{\rm O}$ nanofluids for stretching sheets rise as the heat source parameter $(S>0)$ is increased. From these findings one can see that ${\rm Cu}{-}{\rm H}_{2}{\rm O}$ has higher velocity curves as compared to Al_2_O_3_–H_2_O.

### Skin friction and heat transfer

The local Nusselt number $\left({{Re}_{x}}^{-1/2}{Nu}_{x}\right)$, or simply the local heat transfer rate and skin friction $\left({{Re}_{x}}^{1/2}{C}_{f}\right)$ are critical industrial parameters. Due to industrial use, the importance of these numbers cannot be disputed.

In addition, the $\left({{Re}_{x}}^{1/2}{C}_{f}\right)$ of ${\rm Cu}{-}{\rm H}_{2}{\rm O}$ nanofluids surface area exceeds that of Al_2_O_3_–H_2_O nanofluids. The $\left({{Re}_{x}}^{-1/2}{Nu}_{x}\right)$, on the other hand, steadily rises as the sheet is stretched. For different estimates of $c,\varepsilon ,M,\phi ,\gamma ,$ and $S$, Figs. 30, 31, 32, 33, 34, 35, 36, 37, 38, 39, 40, 41, 42, 43, 44, 45, 46, 47, 48, 49, 50, 51, 52 and 53 illustrate the fluctuation of the decreased $\left({{Re}_{x}}^{1/2}{C}_{f}\right)$ and $\left({{Re}_{x}}^{-1/2}{Nu}_{x}\right)$ towards $\lambda$. Stretch parameter $c$ is presented in Figs. 30, 31, 32 and 33 to demonstrate how it affects $({{Re}_{x}}^{1/2}{C}_{f})$ and $\left({{Re}_{x}}^{-1/2}{Nu}_{x}\right)$. The stretching sheet increased $({{Re}_{x}}^{1/2}{C}_{f})$ and $\left({{Re}_{x}}^{-1/2}{Nu}_{x}\right)$ as can be seen from these numbers. The stretching parameter had the greatest impact on $({{Re}_{x}}^{1/2}{C}_{f})$ along the surface and $\left({{Re}_{x}}^{-1/2}{Nu}_{x}\right)$ at the surface, because of which it was the most important parameter. In addition, the increase in the stretching parameter's value further enhances the free convection's impact.

For stretching a sheet, Figs. 34, 35, 36 and 37 demonstrate the influence of nanofluid suction parameter on $({{Re}_{x}}^{1/2}{C}_{f})$ and $\left({{Re}_{x}}^{-1/2}{Nu}_{x}\right)$. Figures 34 and 35 show that the values of the skin friction decrease when the suction parameter is provided. The suction effect at the boundary slows the mobility of nanofluid and decreases the velocity differential along the permeable stretching/shrinking surface. An unanticipated velocity gradient is formed because of the suction emergence, which causes heated fluid motions to approach the wall and reduce buoyancy strengths induced by the strong viscosity effect. To illustrate this, Figs. 36 and 37 shows that when $\varepsilon$ was exposed on a stretching/shrinking surface, the local heat transfer rate increased significantly. It's common for heat transfer rates to improve as $\varepsilon$ increases in size. The thermal boundary layer's thickness was reduced when the suction parameter value was increased, resulting in an increase in the temperature gradient at the surface. When shown in Figs. 36 and 37, the number of local Nusselt points increases gradually as the value of $\varepsilon$ for stretching sheets of two different kinds of nanofluids containing ${\rm Al}_{2}{\rm O}_{3}, {\rm Cu}$ nanoparticles are increased. According to Figs. 34 and 35, ${\rm Cu}{-}{\rm H}_{2}{\rm O}$ nanofluids have much greater skin-friction coefficients for stretching sheets than nanofluids containing Al_2_O_3_–H_2_O. The rate of heat transfers also driven by the suction parameter ($\varepsilon$), which is one of the concerned parameters in this study.

We displayed the skin friction coefficient $({{Re}_{x}}^{1/2}{C}_{f})$ for developing characteristics to better understand fluid friction at the surface of objects $M$ and $\lambda$ (see Figs. 38, 39). Both nanofluids show a rise in $({{Re}_{x}}^{1/2}{C}_{f})$ at the sheet's surface with a growth in $M$ (stretched instance). This effect is amplified when the fluid includes nanoparticles due to drag forces termed as Laurent forces, that generate reluctance in the movement of fluid particles. $\left({{Re}_{x}}^{-1/2}{Nu}_{x}\right)$ plots in Figs. 40 and 41 show the heat transport in relation to $M$ and $\lambda$ at the surface. The $\left({{Re}_{x}}^{-1/2}{Nu}_{x}\right)$ increases in the event of stretching. There are two ways to limit surface heat transfer: raising the magnitude of the Hartmann number and reducing the amount of viscous force that is exerted on the surface.

Figures 42, 43, 44 and 45 show the effects of varying alumina and copper concentrations on the skin friction coefficient $({{Re}_{x}}^{1/2}{C}_{f})$ and local heat transfer $\left({{Re}_{x}}^{-1/2}{Nu}_{x}\right)$. For both ${\rm Al}_{2}{\rm O}_{3}$-water and Cu-water, the skin friction coefficient increases as a function of increasing $\phi$, which is seen in the data. As a result of the increased fluid viscosity brought on by the increased volume fraction of nanoparticles, the skin friction coefficient was improved. Furthermore, Figs. 42 and 43 show that as the sheet shrinks, a rise in increases the values of the skin portion. Figures 44 and 45 illustrate that when the volume percentage of nanoparticles increases in both conventional fluids (${\rm Al}_{2}{\rm O}_{3}$-water and Cu-water), the distinctive pattern of heat transmission $\left({{Re}_{x}}^{-1/2}{Nu}_{x}\right)$ changes. As the mass of the nanoparticles grows via aggregation, the efficiency of thermal conductivity rises, affecting the rate at which the working fluid transfers heat. Thermal conductivities may rise because of the nanoparticles' increasing temperature and disturbed connection because of internal heat generating impacts.

Figures 46 and 47 show the changes in $({{Re}_{x}}^{1/2}{C}_{f})$ as a function of different heat source parameter $S$ values vs $\lambda$. The skin friction coefficient upsurges with increasing values of heat source.$S$ the heat source parameter with $\lambda ,$ influences $\left({{Re}_{x}}^{-1/2}{Nu}_{x}\right)$, as seen in Figs. 48 and 49. There is a reduction in $\left({{Re}_{x}}^{-1/2}{Nu}_{x}\right)$ as a function of the heat source parameter $S$ with $\lambda$ as indicated in this data set. In situations when the heat source effect is greater, a larger thermal boundary layer reduces the pace at which heat is transmitted. As has been stated before, the existence of the heat production parameter $S$ has the potential to raise both the thermal state of the fluid and the temperature distribution close to the surface. As the temperature goes up, the thermal boundary layer gets thicker and heat transfer at the stretching/shrinking surface goes down. This causes more heat to be pushed to the surface, which is called "induced flow".

Figures 50 and 51 show the skin friction coefficient $({{Re}_{x}}^{1/2}{C}_{f})$ profiles for both nanofluids when a thermally stratified parameter $\gamma$ with $\lambda$ is varied. It has been noticed that $({{Re}_{x}}^{1/2}{C}_{f})$ drops as $\gamma$ increases. Temperature-dependent viscosity parameter, on the other hand, causes maximal changes in local skin friction coefficients with stratification at higher values see Figs, 50 and 51. Figures 52 and 53 show $\left({{Re}_{x}}^{-1/2}{Nu}_{x}\right)$. Temperature variations at various levels of the boundary layer are referred to be “thermally stratified”. Temperature stratification causes freezing liquid to condense at the base of the structure, which reduces kinetic energy and lowers $\left({{Re}_{x}}^{-1/2}{Nu}_{x}\right)$ as temperature stratification increases. Thermal stratification has the characteristic of rising Nusselt number, as seen in Figs. 52 and 53, which is proportional to heat transmission over the moving fluid. As it turns out, the maximum local heat transfer rate can be determined at higher temperatures.

For different physical parameters and nanoparticle volume fractions, we have found a reliable shrinking sheet solution. This study does not address the issue of sheets that are prone to shrinkage due to an unstable solution. For an unstable shrinking sheet solution, it is possible to go farther.

## Conclusion

We studied the impact of nanoparticles aggregation on mixed convective stagnation point flow of ${\rm Al}_{2}{\rm O}_{3}$ and Cu nanofluid with water as base fluid past permeable thermally stratified stretching vertical surface in presence of MHD, heat source and suction. It was used to numerically solve and compute the modified nonlinear ordinary differential equations (ODEs) with altered boundary conditions using the Runge Kutta Fehlberg (RKF) and the shooting approach in Mathematica software. The effects of several regulating factors, such as suction, the nanoparticle volume fraction, magnetic parameters, thermal stratification, heat source, and stretching/shrinking, were explored. Suction $\varepsilon$ and buoyancy $\lambda$ may be used to solve this problem within a particular range in the current study. In both opposing and aiding flow conditions, an increase in heat transfer rate may be seen by including nanoparticle volume fraction and suction. The buoyancy parameter is useful to boost the velocity in the x-direction near the free stream. A few meters away from the melting wall, there is a noticeable reduction in the temperature distribution throughout the flow because of the increased buoyancy. The addition of nanoparticles to the surface resulted in an increase in heat transmission. The ${\rm Al}_{2}{\rm O}_{3}$-water nanofluid has less friction and heat transfer than the Cu-water nanofluid. For heat transmission to be enhanced, the kind of nanofluid is critical. The opposing flow scenario exhibited a greater local heat transfer rate but a lower skin friction coefficient than the assisting flow scenario. The boundary layer separation may be maintained if copper volumetric concentrations grow. The factors of suction and stretching are increasing, which enhances the passage of heat. The local Nusselt number and skin friction coefficient rises in response to an increase in the magnetic parameter $M{^{\prime}}s$ intensity. Thermally stratified parameters boost velocity curves while decreasing temperature profiles. Thermal stratification reduces the rate of heat transmission and the coefficient of local skin friction. The velocity and skin friction profiles of the models with aggregation are greater than those of the homogeneous model. Other researchers from a variety of backgrounds (mathematics, mechanical, and physics) might benefit from this study, which focuses on how to alter (increase or decrease) the heat transfer rate by changing the parameters or the computational capabilities. It is just the combination of copper–water and alumina-water that is relevant to the current findings. To get to the desired result, other researchers may use different classical nanofluid, hybrid nanofluids, tri-hybrid nanofluids, or other physical characteristics.

## Data Availability

The dataset used during the current study are available from the corresponding author on reasonable request.
