# Author Correction: MHD mixed convective stagnation point flow of nanofluid past a permeable stretching sheet with nanoparticles aggregation and thermal stratification

**DOI:** 10.1038/s41598-022-26885-6

**Published:** 2022-12-29

**Authors:** Zafar Mahmood, Sharifah E. Alhazmi, Awatif Alhowaity, Riadh Marzouki, Nadir Al‑Ansari, Umar Khan

**Affiliations:** 1 Department of Mathematics and Statistics, Hazara University, Mansehra, Pakistan; 2 Mathematics Department, Al‑Qunfudah University College, Umm Al-Qura University, Mecca, Kingdom of Saudi Arabia; 3 Department of Mathematics, College of Science and Arts at Alkamil, University of Jeddah, Jeddah, Saudi Arabia; 4 Chemistry Department, College of Science, King Khalid University, Abha, 61413 Saudi Arabia; 5 Chemistry Department, Faculty of Sciences of Sfax, University of Sfax, 3038 Sfax, Tunisia; 6 Department of Civil, Environmental and Natural Resources Engineering, Lulea University of Technology, 97187 Lulea, Sweden

Correction to: *Scientific Reports* 10.1038/s41598-022-20074-1, published online 26 September 2022

The Acknowledgments section in the original version of this Article was omitted. The Acknowledgments section now reads:

“The authors extend their appreciation to the Deanship of Scientific Research at King Khalid University for funding this work through the research group under grant number R.G.P.2/175/43.”

The original Article has been corrected.

